# Understanding Topochemical Microcrystal Conversion in Molten Salt to Produce High-Aspect-Ratio Perovskites Particles

**DOI:** 10.1021/jacs.6c06773

**Published:** 2026-07-25

**Authors:** Ruxue Yang, Shitong Zhou, Berenice Bulteel, Lorna Sinclair, Emma Bryan, Jacob Lewis-Fell, Stefan Michalik, Christina Reinhard, Genoveva Burca, Florian Bouville

**Affiliations:** † Centre for Advanced Structural Ceramics, 4615 Imperial College London, London SW7 2AZ, U.K.; ‡ Diamond Light Source, 5292 University of Manchester at Harwell, Harwell Campus, Didcot OX11 0DE, U.K.; § Faculty of Science and Engineering, The University of Manchester, Oxford Road, Manchester M13 9PL, U.K.; ∥ Imperial College London, London SW7 2AZ, U.K.; ⊥ Diamond Light Source Ltd, Harwell Science and Innovation Campus, Didcot OX11 0DE, U.K.; # Rutherford Appleton Laboratory, ISIS Pulsed Neutron and Muon Source, Harwell Campus, Didcot OX11 0DE, U.K.

## Abstract

Highly anisotropic platelet-shaped particles can be used to enhance both structural and functional applications. Anisotropic perovskites are especially in recent focus for their functional properties, including opto- and piezo-electric. Topochemical microcrystal conversion (TMC) in molten salt has been established as a scalable and robust way to fabricate anisotropic perovskite particles from templates. However, TMC often provides a low degree of control on the particle size and aspect ratio. The control over the particle shape is key to the crystallographic texture and mechanical properties of the final material. A comprehensive examination of the full reaction process is still lacking, leaving the underlying reaction mechanisms in large part unresolved. In this work, we employed in situ time-resolved synchrotron X-ray diffraction to monitor the TMC of Bi_0.5_Na_0.5_TiO_3_ (BNT) from Bi_4_Ti_3_O_12_ (BiT) templates under varying TiO_2_ deficiencies and uncovered new insights into the nucleation and growth mechanisms. We demonstrate that control over nucleation and initial anisotropic crystallite growth continuously dictates the final BNT particles’ morphologies. Based on these new insights, we produce BNT platelets with a 1.2- ∼2.1-times higher median aspect ratio than previously reported. In addition, we fabricated thin (170 nm) Bi_0.5_Na_0.5_TiO_3_–BaTiO_3_ platelets with a median and 90th percentile aspect ratio of up to 17 and 41, 1.4- and 2.9-fold higher than that of BiT templates (∼12 and 15), respectively, further ascertaining our growth model and its applicability to other BNT-based compositions. Our results expand our knowledge of the TMC process and open the way to producing high-aspect-ratio and quality perovskite platelets.

## Graphical abstract

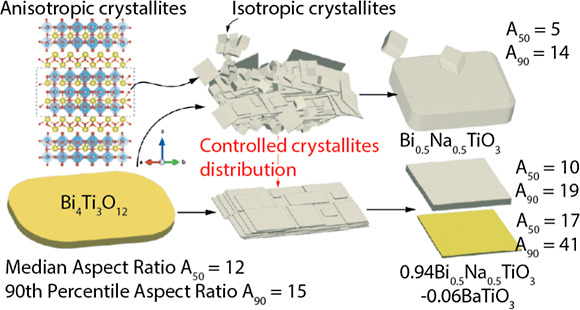

## 1 Introduction

Anisotropic particles are central to control and enhance properties in both structural and functional applications, starting from structural particulate composites^1^ all the way to metal-ion battery and capacitors,^2,3^ catalytic supports,^4^ or piezoceramics.^5^ The properties that these anisotropic particles impart to materials depend not only on their crystal structures and compositions but also on their morphology. The aspect ratio of rods dictates the stiffening in particulate composites,^1^ while at the extreme 2D materials made from platelets of a few atomic layers, including graphene,^6^ MXenes,^7^ and recently 2D perovskites,^8^ present exotic functional properties.

The anisotropic crystal growth, particle shape, and aspect ratio govern the relative fraction of specific crystallographic planes, thereby ultimately changing functional performance. For instance, in perovskite-based platelets, preferentially exposed flat surfaces can promote photocatalytic reactions,^9^ serve as a substrate for thin film growth,^10^ gain medium in laser and optoelectronic devices,^11^ and in flexible sensors.^12^ Bulk materials can use perovskite platelets as a template to influence the distribution of crystallographic orientation.^13^ This concept relies on having anisotropic templates that can be aligned using different types of fields and that will lead to textured samples. This approach led to increased performance in thermoelectric^14,15^ and electrocaloric devices,^16^ piezoelectric constant^17^ and electro-strictive behavior in transducers or actuators,^5^ and multilayered capacitors with higher breakdown strength and energy density.^18^ More recently, the use of Bi_0.5_Na_0.5_TiO_3_ (BNT) platelets to fabricate piezoceramics provided a boost in strength, toughness, and so fatigue resistance without decreasing the piezoelectric performance.^13^

All these property enhancements are built upon the aspect ratio of the platelets used, and higher aspect ratio or more controlled morphology would further these improvements. Perovskites are cubic crystals and thus do not lend themselves to form anisotropic particles.^19–22^ At present, topochemical microcrystal conversion (TMC) is one of the most robust and scalable methods to synthesize perovskite platelets. It relies on converting platelets formed from an anisotropic lattice, mostly Aurivillius phase Bi_4_Ti_3_O_12_ (BiT) templates, into a perovskite while retaining the template’s anisotropic morphology within the molten salt.^19,21–24^ This robust process was developed and used for a few decades now for several compositions, including Bi_0.5_Na_0.5_TiO_3_ (BNT),^21,22,24,25^ BaTiO_3_,^19,26^ SrTiO_3_,^27^ NaNbO_3_,^19^ and PbTiO_3_ platelets.^19^ The mechanisms were described in the seminal paper by Poterala et al.^19^ using ex situ XRD, SEM, and TEM: the conversion from one lattice to another is done with little distortion of the crystal and thus preserves the template morphology and multiple particles grow within the template, forming a mesocrystal that finally turns into a monocrystal.^19^

Although these mechanisms generally fit with the platelet synthesis results,^26,28^ several unexpected features remain, including the presence of equiaxed crystals^21,24,25,29^ or deviations in platelet size and aspect ratio from the template.^21,22,24,25^ These suggest that the existing mechanistic framework is incomplete, calling for further investigation for the mechanisms of templated crystal growth in a fluid medium. The main ones are, especially for BNT platelets, a lack of a direct link between the template aspect ratio and dimension and the final converted ones. Furthermore, according to existing models,^19,26^ the aspect ratio of the template essentially imposes an upper limit on that of the resulting perovskite platelets. Therefore, if the aspect ratio produced via TMC can exceed that of the template limitation, it would constitute an extension to current theoretical frameworks and potentially open new routes to fabricate higher-aspect-ratio perovskite platelets.

Among perovskite candidates, plate-like lead-free (BNT)-based powders enable highly textured ceramics with promising electro-strain, energy storage density, oxide-ion conductivity and exhibit potential for electrocaloric and photoelectric applications. While various reaction parameters have been investigated, such as the Na_2_CO_3_ excess^24,25^ or temperature,^21,24,29^ once the synthesis proceeds, each recipe yields an essentially similar aspect ratio with limited room to further adjust it. In a study from Cha et al.,^29^ it was observed that the diameter of the reagent TiO_2_ particles exhibited a direct positive correlation with the final BNT platelet size, while showing a negative correlation with their aspect ratio, highlighting their role on BNT formation. Therefore, in our work, we employed high-energy, in situ time-resolved synchrotron X-ray diffraction (sXRD) to investigate the complete reaction pathways under varying TiO_2_ deficiencies to further probe the underlying TMC mechanisms. Based on the in situ sXRD, we deduced two distinct reaction pathways depending on the TiO_2_ deficiencies, from BiT (Pathway 1) and Bi_2_Ti_2_O_7_ (BTO) (Pathway 2). We explore the link between the formation of different crystallite populations during the initial conversion and the final platelet morphology by adding ex situ SEM analysis, leading to a modified growth mechanism. The new mechanism explains the observed reduction in aspect ratio and the emergence of undesirable equiaxed grains. Based on this newfound knowledge, we proposed and tested three strategies to tune the BNT platelet morphology continuously. These strategies are then deployed for another BNT-based platelets, the widely used 0.94 Bi_0.5_Na_0.5_TiO_3_-0.06BaTiO_3_ system, where we successfully synthesized platelets with an aspect ratio higher than the template.

## 2 Results and Discussion

We conducted in situ time-resolved sXRD (Figure 1a), then 2D diffraction patterns were azimuthally integrated into 1D diffractograms (Figure 1b) and analyzed by Rietveld refinement to track the evolution of the phases with temperature. The measured temperature had been calibrated using the melting temperature of KCl (Figure S1), and transient effects were neglected given the small crucible size.

**Figure 1:**
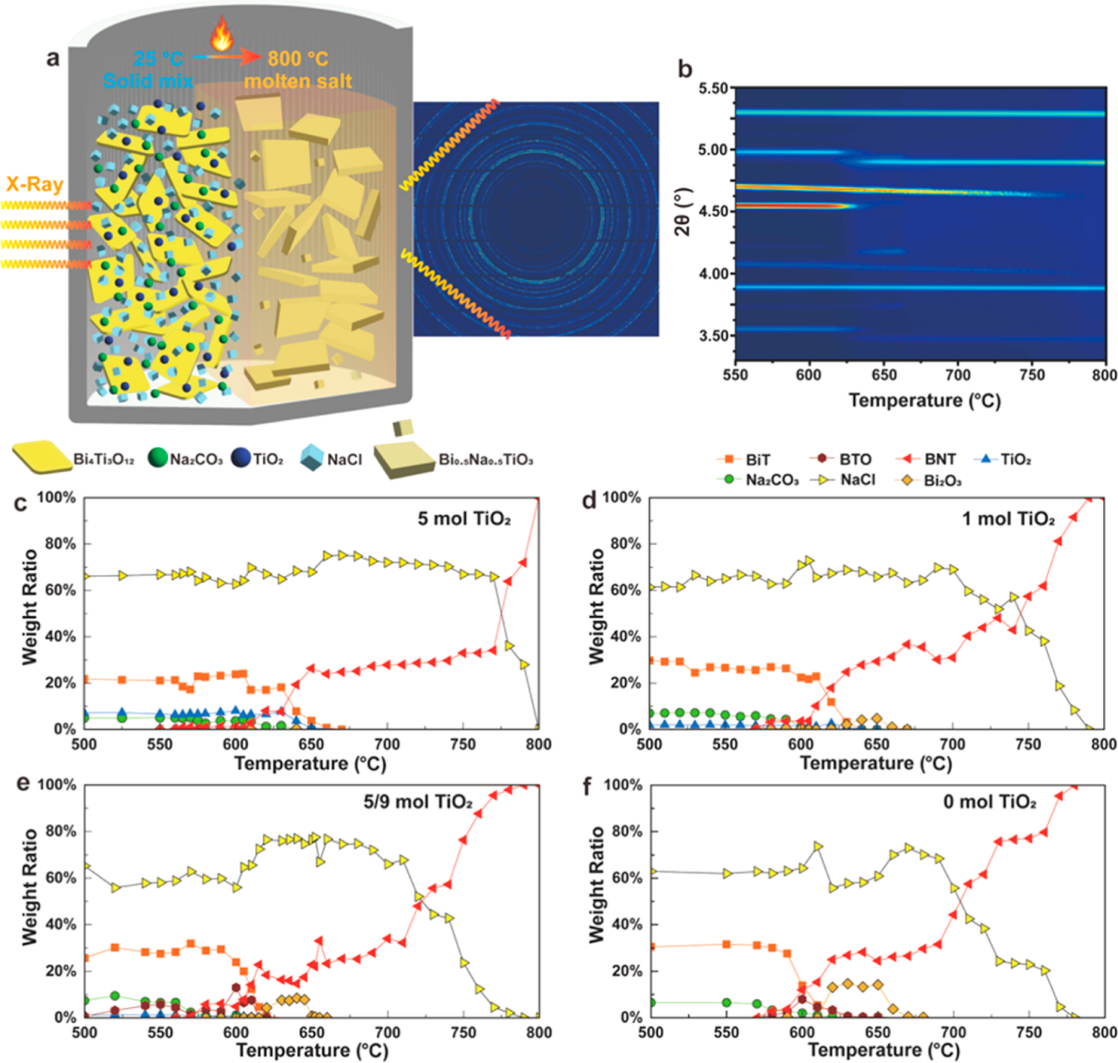
Phase evolution from in situ synchrotron XRD during the reaction from Bi_4_Ti_3_O_12_ (BiT) into Bi_0.5_Na_0.5_TiO_3_ (BNT) in the molten salt. (a) Schematic illustration for the setup for the in situ sXRD with a collected 2D X-ray diffraction image with diffracted rings for the 1 mol TiO_2_ system at 600 °C. (b) Contour plot of the in situ sXRD azimuthally integrated to 1D profiles for the 1 mol TiO_2_ system. Evolution of the ratio of phases as a function of temperature based on Rietveld refinements obtained for (c) 5 mol TiO_2_, (d) 1 mol TiO_2_, (e) 5/9 mol TiO_2_, and (f) 0 mol TiO_2_; the constant Al_2_O_3_ content from the crucible is omitted for clarity.

The evolutions of the phases with varied TiO_2_ deficiencies as a function of temperature are plotted in Figure 1c–f. There are a few common conclusions that stand for all TiO_2_ contents systems. BNT formed between 560 and 675 °C independently on the TiO_2_ content, and its mass fraction remained stable above 675 °C, indicating that the reaction was completed prior to the melting of pure NaCl (∼780–800 °C). Based on the NaCl/Na_2_CO_3_ phase diagram,^30^ around 19–32% of the volume of all reagents became liquid between 575 and 675 °C. This proves that the conversion from BiT to BNT took place with a limited amount of molten salt instead of fully immersed in molten salt.

The reaction pathways change significantly with varying TiO_2_ deficiency, highlighting the critical role of TiO_2_ in dictating the phase evolution. With increasing TiO_2_ deficiency from 5 to 0 mol (Figure 1c–f), Bi_2_O_3_ appeared in progressively larger amounts between 620 and 650 °C, from 0 wt % (5 mol) to 14.8 wt % (0 mol) (Figure S2). We further confirmed that only Bi_2_O_3_ and BNT can be found after washing (Figure S3), establishing Bi_2_O_3_ as a byproduct.^19,26^ Under a severe TiO_2_ deficiency of 5/9 and 0 mol, another compound, Bi_2_Ti_2_O_7_ (BTO), was present over the whole temperature range where BNT forms, at 500–615 °C, 570–640 °C, for 5/9 and 0 mol respectively, reaching 7–15 wt % and was absent after cooling (Figure S3). Therefore, at least two distinct pathways from BiT to BNT can be observed. Pathway 1: BiT → BNT for 5/9 ≤ TiO_2_ ≤ 5 mol; Pathway 2: BiT → BTO → BNT for 0 ≤ TiO_2_ ≤ 5/9 mol. We propose that the following possible reactions are present for Pathways 1 and 2:

For Pathway 1

$$Bi_{4}Ti_{3}O_{12}+\left(0.25x+0.75\right)Na_{2}CO_{3}+xTiO_{2}\to \left(x+3\right)Bi_{0.5}Na_{0.5}TiO_{3}+\left(1.25-0.25x\right)Bi_{2}O_{3}+\left(0.25x+0.75\right)CO_{2}↑\;with\;1\leq x\leq 5$$ 1

For Pathway 2

2 $$2Bi_{4}Ti_{3}O_{12}\to 3Bi_{2}Ti_{2}O_{7}+Bi_{2}O_{3}$$

$$2Bi_{2}Ti_{2}O_{7}+Na_{2}CO_{3}\to 4Bi_{0.5}Na_{0.5}TiO_{3}+Bi_{2}O_{3}\;with\;1\leq x\leq 5/9$$ 3

After detailing the different reaction pathways and associated phase evolution, we analyzed the in situ synchrotron XRD data to gain insight into the nucleation and growth of BNT crystallites.

For Pathway 1, BNT is mainly generated from BiT. Beyond phases amount evolution, in situ sXRD can also be used to extract information on particles’ size and shape using the evolution of relative peak intensities and widths. The Lotgering factor, used to quantify crystallographic texture,^31^ can be used in this context to quantify a possible enhanced or decreased presence of specific planes. As we do not expect the presence of preferential particle orientation during the synthesis, a nonzero Lotgering factor would highlight a larger presence or absence of specific planes and thus anisotropic growth. The *f*_(*hk*0)_ for the 5 and 1 mol TiO_2_ system started at around 80–95% (Figure 2a), whereas *f*_(00*l*)_ was negative across the 575–675 °C range (Figure 2b), confirming the strong anisotropic growth favoring the presence of the prismatic plane. BiT has an Aurivillius structure with a (*Bi*_2_ *O*_2_)^2+^(*Bi*_2_ *Ti*_3_ *O*_10_)^2–^ super lattice (Figure 2c). This crystal is composed of alternating (*Bi*_2_ *O*_2_)^2+^ oxide layers and pseudoperovskite (*Bi*_2_ *Ti*_3_ *O*_10_)^2–^ layers stacked alternately along the ⟨00*l*⟩ direction.^32^ Within the BiT pseudoperovskite layer, the TiO_6_ octahedra spacing is 4.29 Å along ⟨00*l*⟩ and 3.81–3.90 Å along ⟨*hk*0⟩, similar to the spacing in BNT with 3.90 Å (Figure 2d) at 550–675 °C. Therefore, the lattice mismatch along the ⟨00*l*⟩ exceeds 10%, indicating that the interface is necessarily incoherent during the nucleation and growth of BNT crystallite in BiT. The mismatch along ⟨*hk*0⟩ is small, requiring only limited strain accommodation and subsequent preferential nucleation and growth. In addition, the (*Bi*_2_ *O*_2_)^2+^ layer cannot directly transform into a BNT lattice and thus acts as a barrier, suppressing cross-layer growth of BNT along the BiT ⟨00*l*⟩ direction. This structural similarity enables BNT to nucleate and grow along the BiT pseudoperovskite plane, leading to faster lateral growth before ∼675 °C and thin flake-shape anisotropic crystallites (Figure S4a) and to a high *f*_(*hk*0)_ and low *f*_(00*l*)_. Similar lattice lateral nucleation phenomena were also reported in the Bi_4_Ti_3_O_12_-templated TMC for BaTiO_3_ platelets with the TEM.^19,26^ After 675 °C, the anisotropic growth progressively decreased until the Lotgering factors converge to zero at the point of complete conversion to BNT.

**Figure 2:**
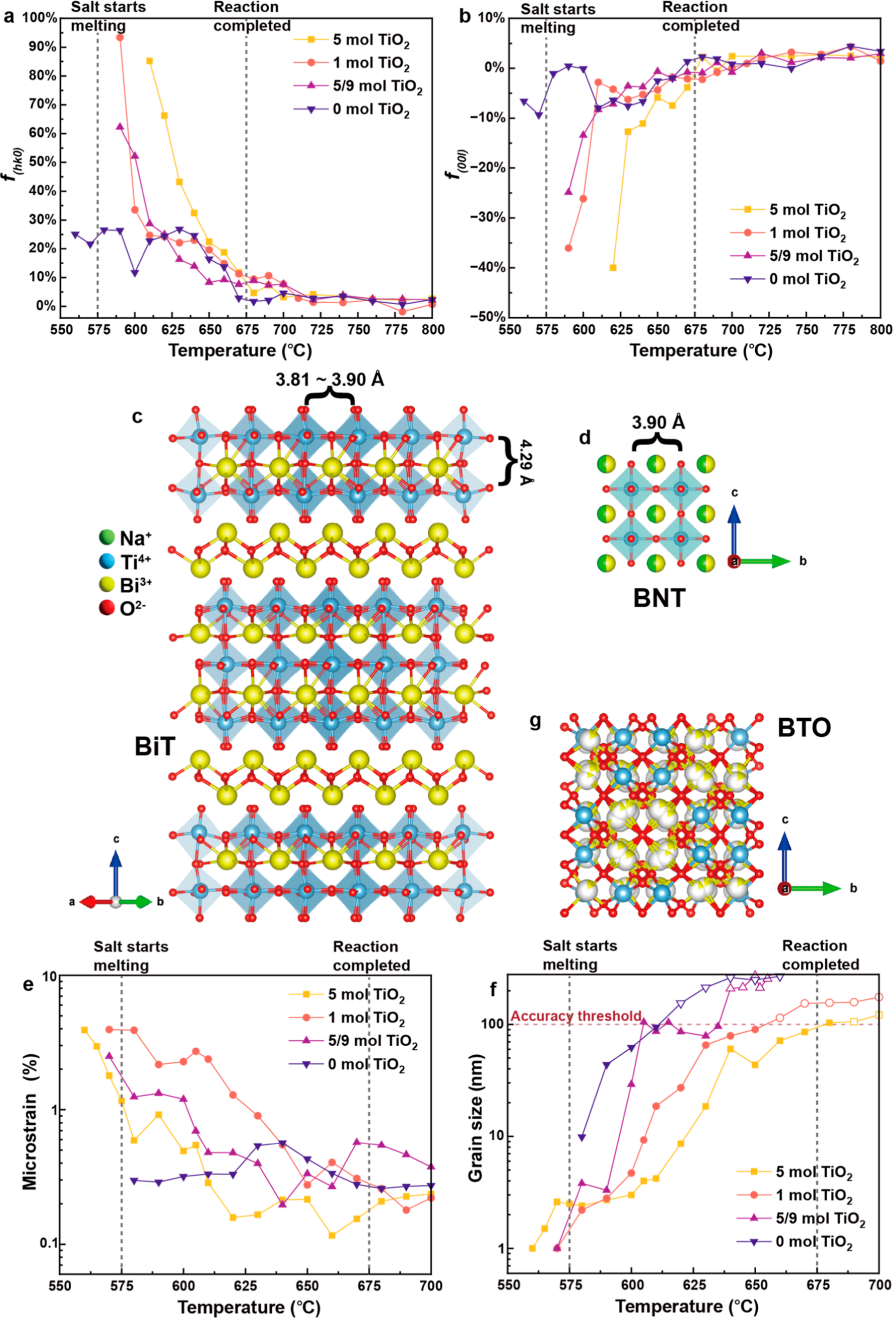
BNT crystallites’ nucleation and growth process reconstructed from in situ synchrotron XRD during the reaction. Evolution of the Lotgering factor for (*hk*0) (a) and (00*l*) (b) as a function of temperature. Lattice schematic for (c) Bi_4_Ti_3_O_12_ orthorhombic phase and (d) Bi_0.5_Na_0.5_TiO_3_ cubic phase. Evolution of the BNT microstrain (e) and crystallite size (f) with a hollowed symbol indicating the data out of the accurately measurable size range according to previous research^35^ and as a function of temperature from the Rietveld refinement. The schematic illustration of the Bi_2_Ti_2_O_7_ lattice (g) cubic phase.

Simultaneously to the anisotropic growth, microstrains initially reached ∼3–4% but dropped to 0.2–0.5% for TiO_2_-contained systems between 550 and 625 °C (Figure 2e). These microstrains are consistent with the BNT growth within the pseudoperovskite layers to accommodate the difference between the TiO_6_ octahedra in the Aurivillius phase and the cubic perovskite phase. Over the same temperature range, all grain sizes started between 1 and 10 nm but increased up to 100 nm and higher (Figure 2f) at ∼700 °C as their microstrains decreased by nearly 2 orders of magnitude to reach 0.1–0.17%. Notably, the 1 mol TiO_2_ sample exhibited the lowest microstrain level between 700 °C and 800 °C, suggesting a transition involving less distortion.

The (*Bi*_2_ *O*_2_)^2+^ oxide layer in BiT will form free Bi_2_O_3_ in the medium, which explains the presence of free Bi_2_O_3_ observed after cooling. Bi_2_O_3_ dissolved into the medium at temperature above 675 °C and can further react with residual TiO_2_ and Na_2_CO_3_, forming a second source of BNT that does not grow from BiT.

4 $$Bi_{2}O_{3}+4TiO_{2}+Na_{2}CO_{3}\to 4Bi_{0.5}Na_{0.5}TiO_{3}+2CO_{2}↑$$

These results imply that secondary BNT forms on TiO_2_ particles, producing equiaxed isotropic crystallites (Figure S4b,c) with size inherited from the TiO_2_ particles.

For Pathway 2, the BTO-intermediated process hints at a different nucleation and growth mechanism at 0 mol TiO_2_ and partially at 5/9 mol TiO_2_ systems. The growth of the BNT crystallite in the 0 mol TiO_2_ system was more isotropic, with *f*_(*hkl*)_ values ranging from 20% to 30% (Figure 2a) within 560–675 °C, 63%–79% smaller than that of the 5 mol TiO_2_ system. In addition, in the 0 mol TiO_2_ system, the strains remained around 0.2%–0.3% compared with 2%–0.4% of 5 and 1 mol of TiO_2_. The BTO adopts a pyrochlore structure with a highly symmetric and isotropic *Fd* 3̅ *m* space group (*a* = 10.317 Å, Figure 2g).^33,34^ The large difference between BTO and the BiT perovskite layer suggests that BTO can only nucleate with no coherency with the host BiT lattice, leading to less microstrain in the newly formed phase.

BTO is known to be metastable near 600 °C and destabilized above 650 °C.^33^ BTO is present in quantities around 1–13 wt % over 575–650 °C where BNT also forms. As an intermediate, BTO provides an isotropic lattice template for subsequent BNT nucleation. A nonzero *f*⟨_*hk0*_⟩ (20–30%, Figure 2a) in the 0 mol TiO_2_ system suggests that some anisotropic BNT crystallites may still nucleate directly from BiT, as in Pathway 1. We provide additional ex situ synthesis results where we increase the Na_2_CO_3_ amount from 30 to 90 mol % at 5/9 and 0 mol TiO_2_. Only small equiaxed BNT grains are obtained (Figure S5), suggesting that a low Ti:Na ratio favors Pathway 2 as well. The transition between Pathway 1 and Pathway 2 appears influenced by the Ti^4+^:Na^+^ molar ratio. Higher ratios (≥0.25) favor Pathway 1, whereas lower ratios (≤0.14) promote BiT-to-BTO conversion (Pathway 2). In the 5/9 mol TiO_2_ system with 30 mol % excess Na_2_CO_3_, both pathways coexist.

With the identification of two pathways and growth modes across the TiO_2_ composition range to form BNT anisotropic and isotropic crystallites, we now analyze how these populations of crystallite evolve after TMC during subsequent growth and dictate the final powder morphology.

SEM images of final BNT platelets after isothermal holding are shown in Figure 3a–d. Reducing TiO_2_ from 5 to 1 mol yielded better-defined platelets exhibiting smoother surfaces, more regular shape, and sharper edges (Figure 3a,b). There were also fewer equiaxed particles compared with the 5/9 and 0 mol (Figure 3c,d). The statistic distributions of aspect ratio consistently exhibited two distinct peaks (Figure 3e–h). The median aspect ratios can be extracted from these distributions, and a bimodal log–normal distribution can be fitted (Table S1) to obtain the fraction of anisotropic particles in Figure 3i. The main percentiles for each dimension of all syntheses are summarized in Table S2. As the TiO_2_ content decreased from 5 to 1 mol, the distribution patterns showed a decrease in the first peak (Figure 3f), accompanied by the emergence of a dominant second peak associated with a higher aspect ratio. The corresponding fraction of high-aspect-ratio particles increased from 64% to 91%, and the overall median aspect ratio A_50_ increased from 4.9 to 8.4 (Figure 3i). However, reducing TiO_2_ from 1 to 0 mol reversed the trend. The distributions showed a more dominant first peaks associated with lower aspect ratios (Figure 3g,h), with the fraction of high-aspect-ratio particles dropping to 15% and the A_50_ aspect ratio decreasing to 2.1 (Figure 3i). This trend suggests a strong link between the final particle shape distributions and the reaction pathway explored using the in situ sXRD results, as the changes are concurrent with the gradual switch from reaction Pathway 1 to 2.

**Figure 3:**
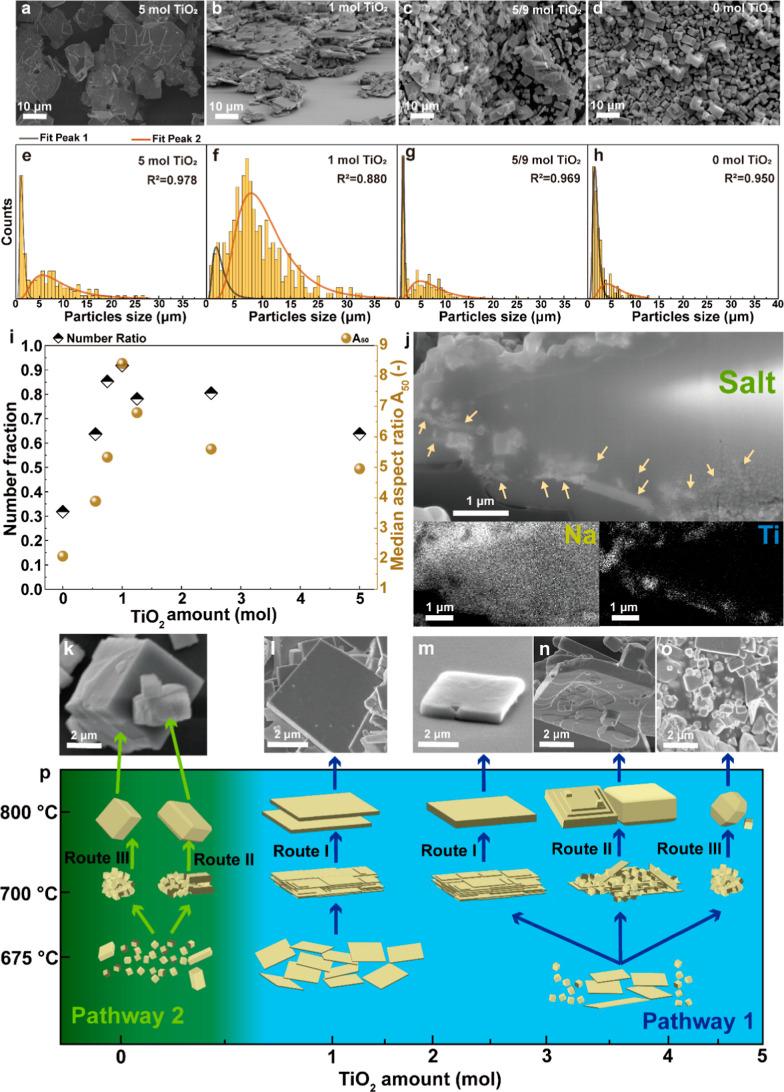
Mechanisms of BNT platelet growth from the crystallite population. SEM images of final BNT platelets morphology evolutions and corresponding grain size distribution of (a,e) 5 mol, (b,f) 1 mol, (c,g) 5/9 mol, and (d,h) 0 mol TiO_2_. Peak 1 corresponds to particles with a lower aspect ratio, whereas Peak 2 corresponds to particles with a higher aspect ratio, both of which are marked in plots. (i) Evolution of the number ratio of the two populations of particles. (j) Platelets median aspect ratio A_50_ as a function of TiO_2_. The representative SEM images of the final equiaxed BNT under various TiO_2_ deficiency (k–o). (p) Schematic illustration of the growth pathway from BNT crystallites to BNT platelets under varied TiO_2_ deficiency.

For all TiO_2_ content, the platelets obtained present both an aspect ratio and size significantly different from that of the BiT template, which presents an aspect ratio A_50_ of 11.9 and a diameter D_50_ of 4.17 μm (Table S2 and Figure S6). Previous TMC studies indicate that BNT nucleates at multiple sites on BiT platelets and that the two phases share some crystallographic coherence, leading to the growth of BNT crystallites that pack together and retain the template shape.^19,20,36^ However, this framework fails to explain several key observations: (a) the lower aspect ratio and different dimensions of the final BNT platelets compared to the BiT template (Table S2 and Figure S6); (b) the irregular shape and rough terraced facet different from BiT (Figure S7a,b) and the bimodal size distribution of BNT (Figures 3e–h and S7c). To further confirm these paradoxes, we synthesized BNT using two sizes of BiT templates. Despite the difference in precursor BiT morphology and size, the resulting BNT powders exhibited similar final morphologies and sizes (Figure S8).

Combining the in situ sXRD with the final particle morphology analysis, we propose a unified description: during conversion from BiT to BNT and before the NaCl melts, two possible reaction pathways exist, Pathway 1 based on the Aurivillius lattice leading to anisotropic BNT crystallites and Pathway 2 mediated by BTO leading to more isotropic BNT crystallites. Partial fragmentation of the template occurs due to the forming crystallites. In addition, in Pathway 1, a side reaction between the released Bi_2_O_3_ and TiO_2_ particles in the medium leads to isotropic crystallite (secondary BNT) formation away from the BiT template. The main driving force for this system to evolve is the reduction of surface energy, making the particles grow and becoming more faceted. The molten salt can accelerate this evolution as it will increase the dissolution–precipitation and mass transport kinetics, as well as promote particle-oriented attachment.^19,37^ Based on this reasoning and supported by the similar-sized BNT obtained from different-sized BiT, we propose that these fragmented crystallites can now, within the molten salt, drift and reform into the particle rather than assembling tightly within the original BiT template shape, denoted as the fragment-drift model. These crystallites subsequently undergo coalescence and growth to form the final BNT crystals. Direct visualization of the evolution of these crystallites is experimentally challenging; thus, in addition to the sXRD, we support this model with ex situ FIB-SEM. We used Focused ion beam (FIB) to cut a solidified salt block from the 5 mol TiO_2_ system which was stopped at 700 °C. These cuts (Figure 3j with additional images in Figure S9a,b) revealed that fragmented BNT crystallites are dispersed in the salt relatively far from each other. These anisotropic crystallites have lengths between 0.2 and 1.2 μm, thicknesses between 0.02 and 0.15 μm, which are significantly smaller than those of the precursor BiT templates. Therefore, it is assumed that partial fragmentation of the template occurs due to the formation of BNT crystallites. In addition to the sXRD and FIB-SEM evidence, we support this model with ex situ SEM and particle size analysis from stopped reactions at different temperature (Figure S9c,d).

The final aspect ratio of the BNT particles can thus be explained by how the population of the nanosized anisotropic/isotropic crystallite evolves in the molten NaCl (Figure 3k–p).

Route I corresponds to the interaction solely between anisotropic crystallites (Figure 3m,p). Oriented attachment of anisotropic crystallites plays an important role to maintain high-aspect-ratio particles. From the anisotropic growth of the crystallites within BiT (Figure 2a,b), the anisotropic crystallites can detach/drift or remain within the BiT template. The speed of the conversion and the presence of molten salt can influence the relative importance of drifting. We arrested the reaction at different temperatures at the 5 mol TiO_2_ system to confirm these different possibilities. From the growth of BNT crystallites in BiT (Figure S4b), some thin crystallites detach while the others stay close, leading to BNT platelets thinner and smaller than BiT (Figure S4b). Since the face and edge of these anisotropic crystallites are similar in composition and crystal structure due to the cubic phase of BNT, the attachment is only dictated by the relative availability of the different surfaces. Therefore, with longer reaction time, there is a higher probability of further face–face attachment, with a random overlap accounting a different and potentially lower aspect ratio than BiT (Figures S4c and S10). Afterward, the adjacent thicker grown platelets can also connect edge-to-edge in the molten salt (Figure S11), expanding their lateral dimensions, increasing the aspect ratio again, and accounting the different diameters than BiT. The final stage of the platelet’s growth consists in a smoothening of the jagged surfaces to decrease the overall surface area of the particles, decreasing the aspect ratio and leading to smooth platelets (Figure 3l,m).

In route II, the isotropic crystallite can also dissolve and reprecipitate or attach to the surface of anisotropic crystallite/assembly of crystallites (Figure 3p), again with a higher availability of the face compared to the edges and thus overall leading to a decrease of the aspect ratio and the presence of rough platelet surfaces (Figure 3n).

In route III, the isotropic crystallites can also dissolve, reprecipitate and attach to other isotropic crystallites, forming isotropic micrometer-sized particles, explaining the bimodal distribution disrupted by equiaxed particles (Figure 3k,o).

We can conclude that moderate TiO_2_ (5–1 mol TiO_2_) deficiency suppresses secondary BNT and increases the fraction of anisotropic crystallites, promoting route I, yielding powders with high aspect ratios and regular shape, whereas heavy TiO_2_ deficiency (0–5/9 mol TiO_2_) promotes the formation of equiaxed crystallites and routes II and III, thereby deteriorating the high-aspect-ratio morphology. Therefore, the local crystallite type and distribution determine the final platelet morphology. To obtain the highest-aspect-ratio BNT particles with the smoothest faces, we need to ensure a TiO_2_ deficiency that (a) suppresses the formation of secondary equiaxed crystallites and (b) leads to the highest possible anisotropic growth from BiT to get the highest fraction of anisotropic crystallites. With the current reaction, the reaction Pathway 1 at 1 mol TiO_2_ starts with the highest anisotropic growth, thus the highest aspect ratio crystallite, and finally leads to the highest aspect ratio micrometer-sized monocrystalline platelets, confirmed by electron backscattered diffraction in Figure S12.

Additional parameters involved in the TMC process were also investigated to further evaluate their influence on the proposed fragment-and-drift model (Figure S13). The reaction temperature strongly affects the growth of the crystallites. Overall, an increase in temperature leads to the growth of platelets from the isotropic particles and smaller platelets through Ostwald ripening. Increasing the molten salt content promotes the dissolution of isotropic crystallites, thereby facilitating the formation of platelets with more regular and smoother surfaces. When increasing time at a constant temperature, the reaction essentially ceases to evolve, in this case, after about 3 h at 800 °C. A change in salt composition also changes the solubility, dissolution kinetics, and surface chemistry, leading to altered fragment morphologies and could be further explored in the future as a possible leverage to change the aspect ratio further.

To provide further evidence to our growth model, we provide ex situ results from arrested synthesis at intermediate temperatures. The first one is the 5 mol TiO_2_ system at 800 °C. The resulting powders present a mix of anisotropic (supposed to follow route I and II) and isotropic particles (route III), with the anisotropic particles reaching a median aspect ratios of *A*_50_ = 14.5, 20% higher than that of the BiT template (Figure S4c and Table S2). Similarly, for the 1 mol TiO_2_ system, stopping the reaction at 800 °C further suppressed the thickness growth and led to a 19% higher median aspect ratio compared with the full reaction, reaching 10.0 (Figure S4d–f and Table S2). In addition, Figure S14 shows a consistent trend in both the 5 and 1 mol TiO_2_ systems. A lower arresting temperature leads to more limited thickness growth, thereby resulting in a higher aspect ratio. In contrast, isothermal holding promotes growth and consequently reduces the aspect ratio. Therefore, arresting reaction and cooling can terminate crystallites growth and enable retention of the platelets in their early high-aspect-ratio state.

Knowing the mechanisms, we can also explain some trends observed in the literature. Cha et al.^29^ achieved more defined platelets by reducing the TiO_2_ particle size, which reduces the isotropic crystallite size and limits Routes II and III by promoting crystallite dissolution over particle attachment. In addition, Li et al.^25^ obtained smoother plate-like grains through a moderate increase in Na_2_CO_3_, which lowers the eutectic point and thus promotes dissolution for crystallites.

Controlling the crystallite distribution through TiO_2_ deficiency offers a simple, direct, and continuously tunable strategy for regulating the final morphology of BNT platelet powders. Nevertheless, this approach also presents inherent limitations. First, the overall powder yield decreases, which is associated with reaction stoichiometry. TiO_2_ deficiency interrupts the complete conversion of the bismuth containing precursor species (BiT or Bi_2_O_3_) and Na_2_CO_3_ to BNT. In the 1 mol TiO_2_ system, 1 mol of BiT can generate 4 mol of BNT, whereas in the 5 mol TiO_2_ system, it can generate 8 mol of BNT. Furthermore, the TiO_2_ deficiency leads to residual Bi_2_O_3_ in the final products. As a result, an additional nitric acid washing step is required during post-treatment to remove residual Bi_2_O_3_, introducing additional synthesis steps and hazards. The arrested-reaction strategy can only be scaled up by limiting the thermal mass of the sample, for instance, by limiting one dimension of the crucible, and so limiting the amount of powder that can be produced each run.

Beyond proposing effective morphology-tuning strategies, 1 mol TiO_2_ BNT powder also showed improved resistivity and suppressed current leakage at elevated temperature, which is beneficial for dielectric applications. Two ceramics were prepared from BNT platelets (5 mol vs 1 mol TiO_2_) with similar density and microstructure (Tables S3 and S4 and Figures S15 and S16). As shown in Figure S17, the 1 mol sample exhibited ∼1.5 orders of magnitude higher resistivity than the 1 mol sample (2.75 × 10^8^ vs 1.12 × 10^10^ Ω cm at 25 °C). Its temperature-dependent permittivity exhibited smoother, much lower losses (0.05) up to 350–400 °C and a narrower, more convergent permittivity peak (1950–2130 above 300 °C). This dielectric augmentation comes from the extra Bi_2_O_3_ in the molten salt medium under the TiO_2_ deficiency, compensating the Bi evaporation and decreasing oxygen vacancies formation at high temperature.^38^

Our newfound knowledge should be applicable across other systems relying on TMC, and we demonstrate that it can be used to obtain high-aspect-ratio particles of the classic morphotropic phase boundary (MPB) composition BNT-BT (94 mol % Bi_0.5_Na_0.5_TiO_3_-6 mol % BaTiO_3_).

As shown in the in situ sXRD patterns (Figure S18a,b), the BNT-BT reaction pathway closely mirrored that of pure BNT, with only a need for a shift of the holding temperature to 850 °C. Like BNT, the BNT-BT system also stabilizes into a symmetric, isotropic cubic phase at 850 °C.^13^ Samples with the reaction stopped at 850 °C confirmed a similar particle growth mechanism based on anisotropic crystallite-oriented attachments like Route I and isotropic crystallites aggregations like Route II (Figure S18c). All these indicate that the nucleation and growth mechanism identified in BNT also applies to BNT-BT. We successfully synthesized BNT-BT platelets powders, varying the TiO_2_ content from stoichiometric with 5.51 mol to substoichiometric with 2.76 mol, both exhibiting consistent platelets morphology. The general theoretical reaction pathway is

5 $$Bi_{4}Ti_{3}O_{12}+\left(0.235x+0.705\right)Na_{2}CO_{3}+\left(0.06x+0.18\right)BaCO_{3}+xTiO_{2}\to \left(x+3\right)Bi_{0.47}Na_{0.47}Ba_{0.06}TiO_{3}+\left(1.295-0.235x\right)Bi_{2}O_{3}+\left(0.295x+0.885\right)CO_{2}↑,2.76\leq x\leq 5.51$$

Energy-dispersive X-ray spectroscopy in SEM (Figure S19a) confirmed the Ba^2+^ incorporation, and Rietveld refinement of XRD (Figure S19b and Table S5) placed both samples within the rhombohedral-tetragonal MPB region. Based on Figure 4a,b, the 5.51 mol TiO_2_ BNT-BT ceramics formed well-defined square platelets (diameter D_50_ 4.75 μm, thickness T_50_ 1.25 μm, and aspect ratio A_50_ 3.81, Figure 4f) but retained a noticeable population of low-aspect-ratio grains (Figure 4e). In contrast, the 2.76 mol TiO_2_ sample presented a pronounced morphological difference: T_50_ thickness showed a 7.2-fold decrease to 0.17 μm with a diameter of 2.63 μm (50% thinner than templates BiT), yielding a 4.4-fold higher median aspect ratio than 5.51 mol TiO_2_ (Figure 4c–e). The thickness value was further confirmed using AFM (Figure S20 and Table S6). The aspect-ratio distribution also broadened, from a single narrow peak, with a maximum aspect ratio observed of ∼20 for 5.51 mol TiO_2_ to multiple peaks spanning aspect ratios of 5–85 in the 2.76 mol TiO_2_ composition. Notably, the 2.76 mol TiO_2_ BNT-BT median and 90th percentile platelets aspect ratio surpassed by 1.4- and 2.9-fold these of the BiT templates, 16.7 vs 11.9 and 41.4 vs 14.5, respectively, and presented a 50% lower median thickness, 0.17 μm vs 0.33 μm, respectively (Table S2). This phenomenon supports again our fragmentation-drift model to describe the different crystallites growth process where the layered nucleated anisotropic BNT-BT crystallites delaminate off from the template matrix and assemble separately later.

**Figure 4:**
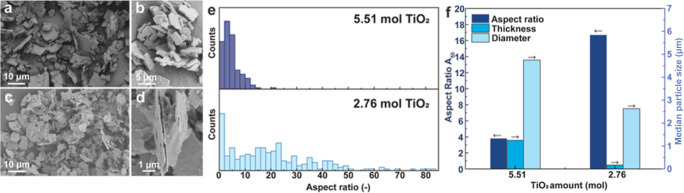
Novel synthesis strategy for BNT-BT high-aspect-ratio powder. SEM images for the BNT-BT morphology synthesized by the TMC method with different TiO_2_ deficiencies of (a,b) 5.51 mol TiO_2_ and (c,d) 2.76 mol TiO_2_. (e) Aspect ratio statistic distribution comparison for BNT-BT platelets powder with 5.51 and 2.76 mol TiO_2_. (f) Statistic median value based on log–normal calculations for BNT-BT powders for aspect ratio A_50_, thicknesses T_50_, and diameters D_50_.

This marked increase in anisotropic morphology and ultrathin platelets could be attributed to the reduction of the number of secondary BNT-BT isotropic crystallites generated from extra TiO_2_ in the TiO_2_ deficiency system, suppressing routes II and III. Compared with pure BNT, the more pronounced increase in the aspect ratio and decrease in thickness observed in the BNT-BT system also reflect intrinsic differences, possibly linked to a slower initial crystallite growth within the BiT brick.

Therefore, the successful application of this strategy to BNT-BT indicates its potential broader applicability to the synthesis of platelet powders in other BNT-based systems.

## 3 Conclusion

In this work, we used time-resolved in situ sXRD to track the full TMC process from BiT into BNT platelets under varying TiO_2_ deficiencies in molten salt. Comprehensive Rietveld refinement resolved two distinct reaction pathways: from Aurivillius phase to BNT (Pathway 1) and BTO-mediated to BNT (Pathway 2). Pathway 1 preserves the layered anisotropy based on the Aurivillius framework, enabling radial growth along ⟨*hk*0⟩ that produces more anisotropic crystallites. Furthermore, with moderate TiO_2_ deficiencies (≥1 mol), less TiO_2_ reacts with Bi_2_O_3_ and free Na^+^ to form secondary isotropic crystallites. Under heavy TiO_2_ deficiencies leading to Pathway 2, we observed a more isotropic growth of BNT crystallites, arising from the cubic phase BTO intermediate in the reaction. The subsequent monocrystal growth is governed by the local distribution and assembly of these mobile anisotropic and isotropic crystallites in the molten salt, a fragmentation-drift mechanism. The growth proceeds through Ostwald ripening and oriented attachment of anisotropic crystallites. Subsequently, the partially grown platelets thicken through face-to-face attachment, while edge-to-edge assembly expands the lateral dimensions and maintains a high aspect ratio. The presence of isotropic crystallites drives the system toward lower overall aspect ratios. This new mechanism accounts for previously unexplained morphological features, expanding upon currently used theories. While it decreases the reaction yield and introduces additional synthesis steps, we can now use this newfound knowledge to produce high-aspect-ratio BNT-based platelets with various strategies: (1) TiO_2_ deficiencies suppressing isotropic crystallite formation; (2) cooling after fragmentation to limit subsequent growth, leading to BNT platelets with a higher median aspect ratio than the template; and (3) refining the isotropic crystallite size. At a 1 mol TiO_2_ system, these mechanisms yield BNT powder with 91% of smooth faceted platelets with a median aspect ratio A_50_ up to 8.41–10.0 and reduced dielectric loss at elevated temperatures. Interestingly, this TiO_2_ deficiency strategy also applies to the BNT-BT MPB composition, leading to thin BNT-BT (*T*_50_ = 170 nm) platelets with 1.4-fold higher median aspect ratios (*A*_50_ = 16.7) and a 2.9-fold increase in the 90th percentile of the aspect ratio (*A*_90_ = 41.4) compared to the BiT templates. This demonstrates the strong potential of this strategy for other BNT-based and potentially other TMC reaction, effectively providing a new methodology to continuously control the morphology of perovskite platelets.

## 4 Materials and Methods

### 4.1 Bi_4_Ti_3_O_12_ Precursor Templates Powder Molten Salt Synthesis

To realize the BNT topochemical microcrystals conversion, the first step is synthesizing the templates Aurivillius phase Bi_4_Ti_3_O_12_ via Molten salt synthesis (MSS), as the following formula:

6 $$2Bi_{2}O_{3}+3TiO_{2}\to Bi_{4}Ti_{3}O_{12}$$

Bi_2_O_3_ (Sigma-Aldrich, 99.9%, with 3 wt % excess amount used to compensate the Bi:Na ratio, as Bi evaporation during molten salt calcination can disrupt the stoichiometry) and TiO_2_ (Sigma-Aldrich, 99.8%) under molten salt mixture of NaCl (VWR, >99.5%) and KCl (Sigma-Aldrich, >99.0%) (molar ratio 1:1). The mixture and reagents were mixed in a weight ratio of 1:1 in ethanol and ball-milled for 24 h with zirconia milling balls. The well-broken and mixed mixtures were dried and heated at 1030 °C for 2 h in speed of 5 °C/min as reported recipes.^21,24,25,39^

### 4.2 Bi_0.5_Na_0.5_TiO_3_ Platelets Powder Topochemical Microcrystal Conversion under MSS

The TMC is used to converse the BiT lattice into BNT platelets with modulated morphologies under different TiO_2_ deficiencies. BiT precursors were mixed with Na_2_CO_3_ (Sigma-Aldrich, >99.5%), TiO_2_ with a variance amount (Sigma-Aldrich, 99.8%) in NaCl (VWR, >99.5%) in ethanol and magnetically stirred for 48 h, with a weight ratio of reagents to salt of 1:2. Especially, the TiO_2_ amount varied from 5 mol, 2.5 mol, 1.25 mol, 1 mol, 0.75 mol, 0.56 mol, and 0 mol, according to the stoichiometric amount in formula

7 $$Bi_{4}Ti_{3}O_{12}+2Na_{2}CO_{3}+5TiO_{2}\to 8Bi_{0.5}Na_{0.5}TiO_{3}+2CO_{2}↑$$

Therefore, there would be 7 sets of different TiO_2_ amounts for subsequent TMC synthesis, while the amount of Na_2_CO_3_ was consistently set to 1.3 times the stoichiometric value where TiO_2_ was set at 5 mol, corresponding to a 30 wt % excess, to compensate for volatilization during the high-temperature reactions. The mixtures were dried and heated at 800 °C for 3 h at a rate of 3 C/min. The obtained powder mix was washed with deionized water to remove the salt and dried. Then, the mix was washed with nitric acid solution (Sigma-Aldrich, 65 wt %) to remove Bi_2_O_3_. A centrifuge was used to separate BNT powder and nitric acid. BNT powder is washed with deionized water again to remove the nitric acid from BNT powder.

### 4.3 0.94 Bi_0.5_Na_0.5_TiO_3_–0.06 BaTiO_3_ Platelets Powder Topochemical Microcrystal Conversion under MSS

To synthesize the MPB BNT-BT with TMC, BiT precursors were mixed with Na_2_CO_3_ (Sigma-Aldrich, >99.5%), TiO_2_ with various amounts (Sigma-Aldrich, 99.8%), BaCO_3_ (Sigma-Aldrich, ≥99%) in NaCl (VWR, >99.5%) in ethanol and magnetically stirred for 48 h, with a weight ratio of reagents to salt of 1:2. Especially, the TiO_2_ amount varied from 100% and 50% according to the stoichiometric amount in formula [Disp-formula eq5], therefore, leading to 2 sets of different TiO_2_ amounts for subsequent TMC synthesis. The amount of Na_2_CO_3_ was consistently set to 1.3 times corresponding to a 30 wt % excess at each set’s themselves stoichiometry, to compensate for volatilization during the high-temperature reactions. The mixtures were dried and heated at 850–870 °C for 3 h at a rate of 3 °C/min. The same washing process was applied as that of washing BNT.

### 4.4 In Situ Time-Resolved Synchrotron Diffraction Experiment on I12-JEEP at the Diamond Light Source

Using an in situ X-ray diffraction experiment setup on the I12-JEEP Beamline^40^ at the Diamond Light Source allowed us to access the evolution of the particles’ phase and size within the TMC MSS to build the reaction pathway for BNT platelets formation. Cylindrical Al_2_O_3_ crucibles for TGA (shown in Figure S21, outer diameter 3.8 mm, height 10 mm, and thickness 1 mm, NETZSCH) were filled with the sample powder are mounted on an alumina rod and inserted in a split furnace (Severn Thermal Solutions furnace model SF2149B, Figure S15) with a entrance and exit hole to accommodate an incoming and scattered X-ray beam, respectively. This type of Al_2_O_3_ crucible has been tested before extensively to make sure it resists the molten salt reaction over a longer period than necessary for the experiments. The sXRD data were collected continuously using a 2D detector (Pilatus 2 M CdTe) in a transmission geometry, a beam size of 0.5 × 0.5 mm^2^, and an energy of 53 keV. The X-ray energy and experimental geometry (e.g., a beam position, detector tilt, and sample-to-detector distance) were determined by measuring a NIST CeO_2_ powder standard sample at multiple sample-to-detector positions with a defined relative offset along a beam path following Hart et al.’s approach.^41^ During the measurements, diffraction data were continuously acquired with an exposure time of 10 s for each frame, while data storage required an additional 0.1–0.3 s. Therefore, the effective temporal resolution corresponded to one diffraction data set collected every ∼10.1–10.3 s. The total scan duration for each sample was approximately 6–7 h. The collected 2D diffraction images were azimuthally integrated into 1D intensity curves as a function of a scattering angle 2θ using the DAWN software.^42^ The split furnace uses a type K thermocouple to measure the temperature a few millimeters outside the crucible. To access the temperature in the crucible, we calibrated the reading using pure KCl (Sigma-Aldrich, ≥99.0%) melting point of 770 °C using a similar heating rate as the TMC experiments (3 °C/min), with the melting temperature measured as the temperature where the XRD peaks fully disappeared from the diffractogram (Figure S1). We used a linear regression between this melting temperature and room temperature to access the temperature in the crucible as a function of the temperature measured by the thermocouple and use this calibrated temperature for the rest of the study. For BNT, the 5 mol, 2.5 mol, 0.56 mol, and 0 mol TiO_2_ mixture was heated to 800 °C at 3 °C/min in air and held for 3 h, and for BNT-BT, the stoichiometric mixture was heated to 850 °C at 3 °C/min in air and held for 3 h.

### 4.5 XRD Data Refinement and Phases Data Extraction

In addition to the in situ sXRD data, all the other ex situ XRD patterns were collected using a MPD Panalytical XRD. The material of each phase was confirmed first using HighScore (Malvern Panalytical). The instrument parameters were calibrated based on standard LaB6 diffraction patterns. To obtain the phase evolution, both Rietveld refinement and Le Bail refinement were performed using the General Structure Analysis System, II (GSAS-II, Argonne National Laboratory^43^). Initially, Le Bail fitting with fixed scale factors (set to 1.0) was employed to extract rough lattice parameters and microstructural parameters for each phase, ensuring rapid alignment between calculated and experimental data (Rwp = 5–15%). The refinement was then transitioned to full Rietveld refinement, beginning with phase fraction refinement and texturing degrees, followed by sequential adjustment of lattice parameters, grain size, microstrain and phase ratio for each phase, until satisfactory convergence was obtained (Rwp = 2–10%). The refinement was performed over a 2θ range of 3.0–6.2° (or to 7.0°), where all phases exhibited their most intense diffraction peaks. The detailed XRD refinements for 1D diffraction data, fitted pattern, difference curve, and Rwp values across several temperatures are shown in Figure S22. The cell parameters, phase ratios, microstrain, and grain sizes evolving during reactions were extracted based on the refinements. During refinement, an isotropic model was used to fit microstrain and domain size. For all the textured phases, which includes the alumina crucible and BNT at some temperatures, a spherical harmonic model with harmonic order 2–8 was used to refine the texturing. No constraints were applied during the refinement process. The temperature-dependent Lotgering factor *f* was used to evaluate the ⟨00*l*⟩and ⟨*hk*0⟩ texture and was calculated based on related peak intensity integration, as the following equation:^31^

$$f_{(00l)\;or\;\left(hk0\right)}=\frac{P-P_{0}}{1-P_{0}}\left(1\right)$$ 8

$$P=\frac{\sum I_{\left(00l\right)\;or\;\left(hk0\right)}}{\sum I_{\left(hkl\right)}}$$ 9

10 $$P_{0}=\frac{\sum I_{\left(00l\right)\;or\;\left(hk0\right)}^{0}}{\sum I_{\left(hkl\right)}^{0}}$$

The *I*_⟨*hkl*⟩_ means the integrated peak intensity of equivalent crystallographic ⟨*hkl*⟩ direction in the sample and *I*_⟨*hkl*⟩_^0^ integrated peak intensity of equivalent crystallographic ⟨*hkl*⟩ direction in a nontextured sample. Specifically, the raw XRD data obtained for a given composition at a specific temperature were plotted in Origin as one-dimensional intensity–angle profiles. The integrated intensity area, *I*_⟨*hkl*⟩_, of each diffraction peak was then calculated directly by numerical integration, with the corresponding peak regions defined around the 2θ range of 2.0–8.0°. The reference XRD diffraction peaks for standard BNT were taken from ICSD entry data_146190-ICSD, corresponding to the cubic Pm3m BNT phase. I_⟨*hkl*⟩_^0^ was calculated based on its peak intensity integration as well.

### 4.6 Morphologies and Sizes Measurements Using Electron Microscopy Techniques

Platelets morphology grain size and aspect ratio characterization using SEM for already synthesized and washed BNT powder. SEM figures were obtained using a TFS Quanta ESEM and Zeiss Sigma300. The powder size distribution and aspect ratio distribution were extracted from SEM images and ImageJ.^44^ Powder was dispersed in ethanol and dropped on the SEM substrate to make sure the platelets lie parallel on the substrate Multiple SEM images were acquired for each sample. During image acquisition, the images were taken without selecting criteria. For each randomly selected region, two SEM images were collected in the same area: the first image was taken with a top view of the platelets; the second image was obtained with a 70° tilted substrate. In total, 6–10 areas were collected for each sample. For the statistical analysis, SEM images and measurement regions were randomly selected. Particle dimensions were analyzed using ImageJ. To avoid selective sampling bias, size measurements were always conducted over an entire continuous region, ensuring that all particles within the selected area were included in the analysis rather than only isolated individual particles. The diameter *d* was measured from the first top view of the platelets, and then the thickness *t*′ of each platelet was measured manually on the tilted image and corrected to the actual thickness *t* to account for the viewing angle: *t* = *t*′/sin 70°. This method allows us to directly distinguish equiaxed and platelets. The aspect ratio *A* was subsequently calculated from the measured diameter and thickness, using *A* = *d*/*t*. For each data set, approximately 200–500 particles were measured.

FIB cuts show the BNT microstructure. FIB milling was performed in an Auriga CrossBeam, Zeiss instrument at an acceleration voltage of 30 kV, and a current of 2 nA to mill BNT cross sections. FIB cleaning of the cross sections was performed at the same accelerating voltage and a current of 50 pA.

## Supplementary Material



## Notes

Materials and methods, synthetic procedures, AFM measurements, fitting data, temperature calibration, phase weight ratio, XRD patterns, SEM for the BNT systems platelets morphologies, SEM images, fitting parameters, sizes distribution, morphology comparison, BNT powder, BNT morphology additional SEM images, different mode of anisotropic crystallites and isotropic crystallites, SEM and illustration for BNT platelets, SEM and EBSD pattern, SEM images, variations in the median diameter, densities for BNT ceramic, SEM images, grain size measurements, XRD patterns, improved functional properties, BNT-BT platelets TMC synthesis, EDX and XRD, phase ratio and lattice parameters, thickness distribution, statistical summary, in situ synchrotron time-resolved X-ray diffraction setup, and sXRD refinement raw data (PDF)
