# Supplementary material for: Understanding Topochemical Microcrystal Conversion in Molten Salt to Produce High-Aspect-Ratio Perovskites Particles
Source: J Am Chem Soc. 2026 Jul 25;148(30):32065–76. doi: 10.1021/jacs.6c06773 (PMC13450376; doi:10.1021/jacs.6c06773)
Supplement: Supplementary file 1 [file ja6c06773_si_001.pdf]

Understanding topochemical microcrystal conversion in molten salt to produce high aspect ratio perovskites particles

*Ruxue Yang[a], Shitong Zhou[a], Berenice Bulteel[a], Lorna Sinclair[b, c], Emma Bryan[d], Jacob Lewis-Fell[b, c], Stefan Michalik[e], Christina Reinhard[b, c], Genoveva Burca[c, e, f], Florian Bouville[a]**

[a] Centre for Advanced Structural Ceramics, Imperial College London, London, SW7 2AZ, United Kingdom

[b] University of Manchester at Harwell, Diamond Light Source, Harwell Campus, Didcot, OX11 0DE, United Kingdom

[c] The University of Manchester, Faculty of Science and Engineering, Oxford Road, Manchester, M13 9PL, Great Manchester, United Kingdom

[d] Imperial College London, London, SW7 2AZ, United Kingdom

[e] Diamond Light Source Ltd, Harwell Science and Innovation Campus, Didcot, OX11 0DE, United Kingdom

[f] ISIS Pulsed Neutron and Muon Source, Rutherford Appleton Laboratory, Didcot, OX11 0QX, Oxfordshire, United Kingdom

*email: f.bouville@imperial.ac.uk

Supplementary method

Ceramic sample fabrication for electrical test

The BNT platelets powder with 5 mol TiO_2 (reference) and 1 mol TiO_2 (optimum sample) were pressed under 150 MPa uniaxial pressing and further pressed in a cold isostatic press at 250 MPa. These two green bodies were sintered at 1100 °C for 4 hours. The samples were machined out from the sintered disks into round disks with diameters of 20 mm and thickness of 1-2 mm. Electrodes were deposited onto the upper and lower end faces of the disk-shaped specimens as follows: first, a layer of conductive silver paste (FuelCellMaterials, FCM 233006 Ag-I) was applied to both end faces and dried at 120 °C for 20 minutes. An additional coat of silver paste was applied and the samples were fired at 650 °C for 30 minutes to ensure robust adhesion of the electrodes. The frequency and temperature evolution permittivity performance were measured by 1296A Dielectric Interface and analysis software SMaRT (from Solartron analytical) from 25 °C to 500 °C under 10^2 - 10^6 Hz. The ε_{33}^{T} and Loss is calculated based on the formula:

$$\varepsilon_{33}^{T} = \frac{1}{\varepsilon_0} \times \frac{Cd}{A} \ (1)$$

$$C = \frac{-1}{2|Z''|\pi f} \ (2)$$

$$Loss = tan\delta \ (3)$$

where $\varepsilon_0 = 8.85 \times 10^{-12}\ F/m$ is the vacuum permittivity, C is the capacitance calculated based on Electrochemical Impedance Spectroscopy (EIS), d is the sample thickness, A is the sample area, $|Z''|$ is the magnitude of the imaginary part of the impedance in EIS, f is the measuring frequency, δ is the loss angle.

Atomic Force Microscopy (AFM) Measurements

A small amount of powder was ultrasonically dispersed in acetone, drop-cast onto glass slides, and dried before AFM characterization. After drying, the platelets were observed to lie

flat on the glass substrate, as confirmed by the AFM height maps. AFM was carried out in tapping mode on an Asylum MFP-3D instrument using NuNano Scout 350 probes, a scan area of 20 μm and a scan rate of 0.2 Hz. Based on AFM measurements collected from eight randomly selected regions, the thickness distribution of the platelets was statistically analysed and compared with that obtained from the original SEM measurements. The corresponding 10th percentile, median, and 90th percentile values of the platelet thickness were extracted from this distribution.

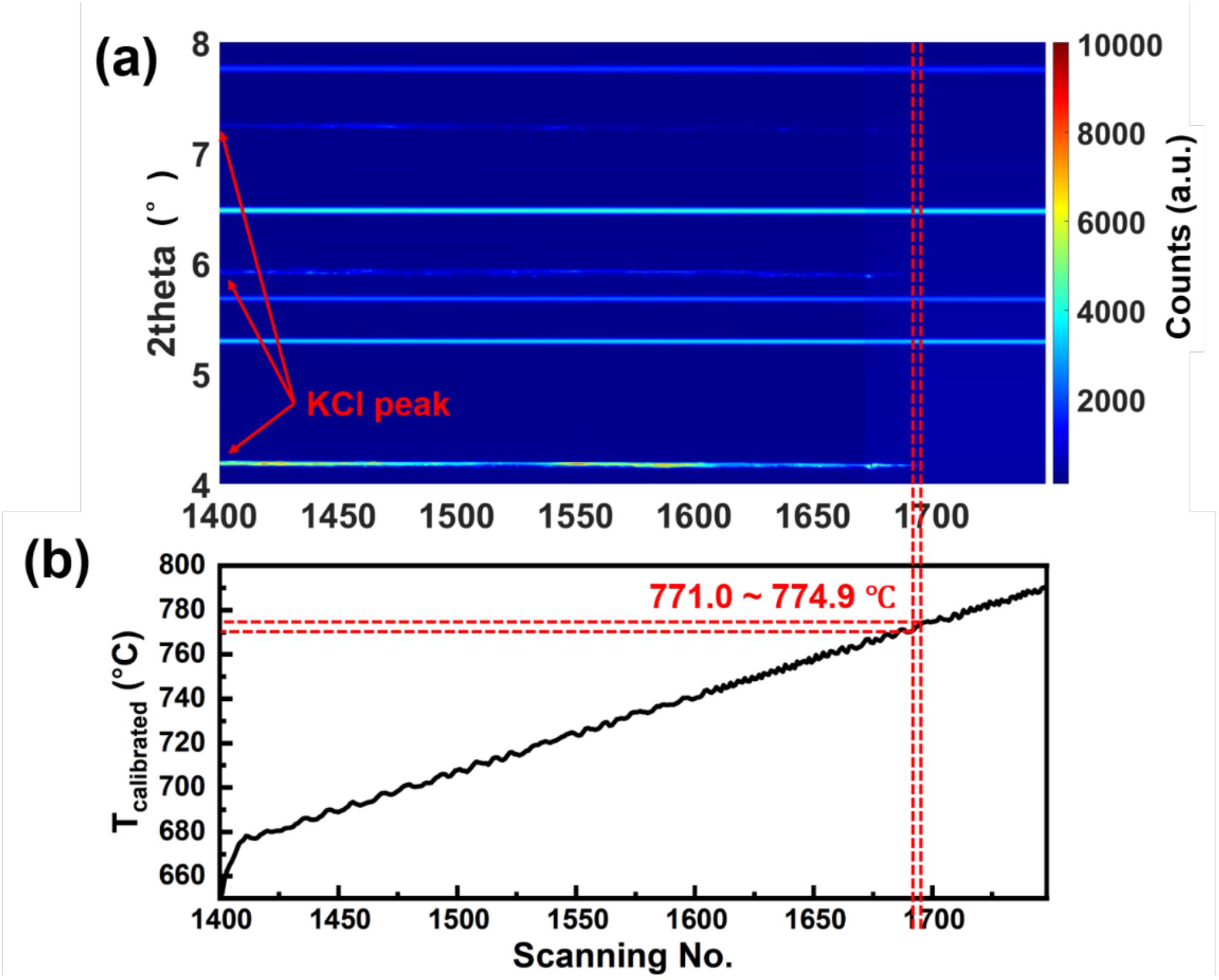

Figure S1. Temperature calibration based on KCl melting in Al_2O_3 crucible. (a) The *in situ* XRD contour plot depends with KCl peaks (marked) and Al_2O_3 peaks. (b) The calibrated temperature as a function of the scanning numbers.

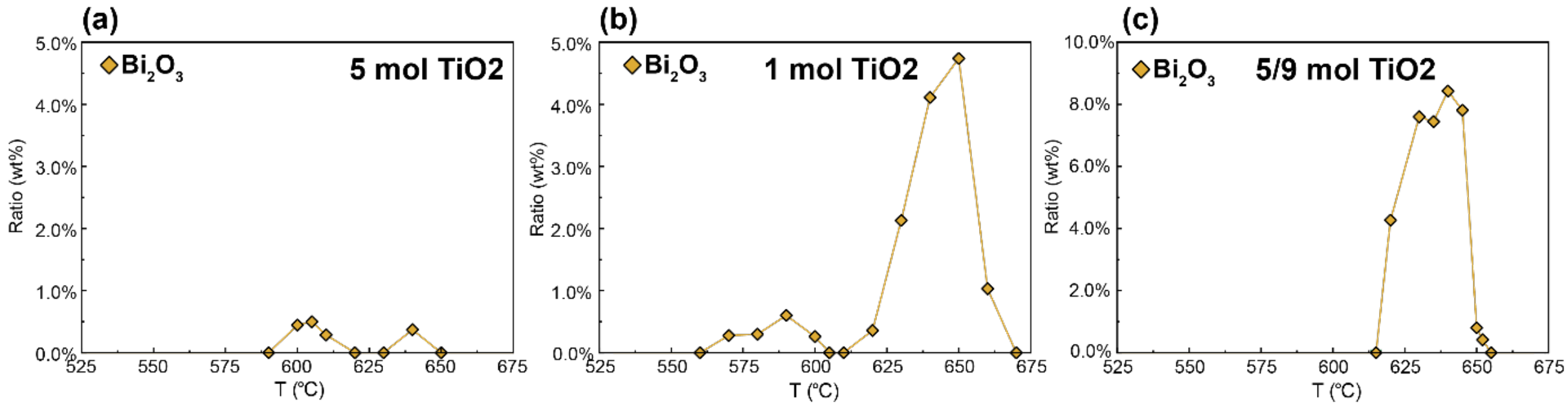

Figure S2. Phase weight ratio for Bi_2O_3 from (a) 5 mol, (b) 1 mol and (c) 5/9 mol TiO_2.

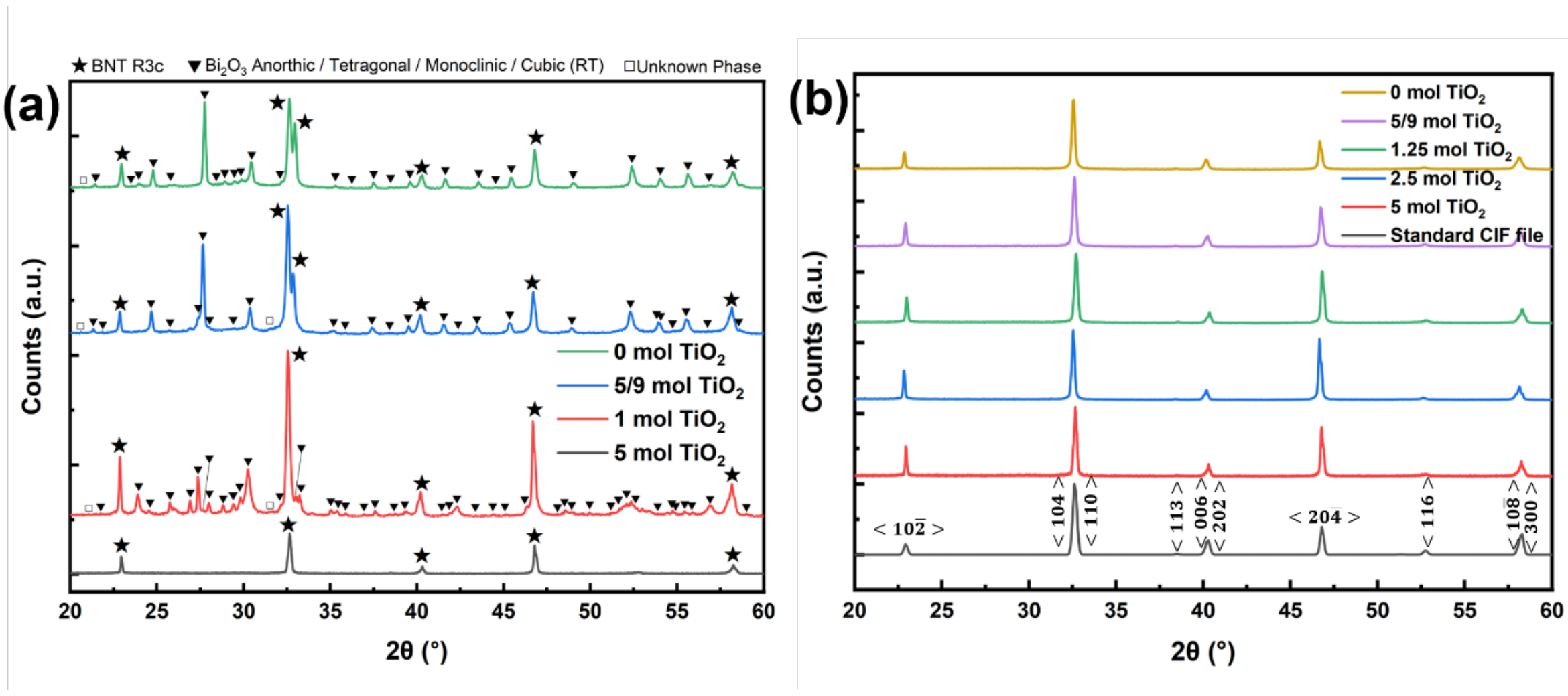

Figure S3. XRD patterns for (a) BNT powder sample synthesized with 5 mol ~ 0 mol TiO_2 amount after water washing to clean out all the water-dissolvable substances; and (b) all BNT powder synthesized after water and nitric acid wash to remove impurities.

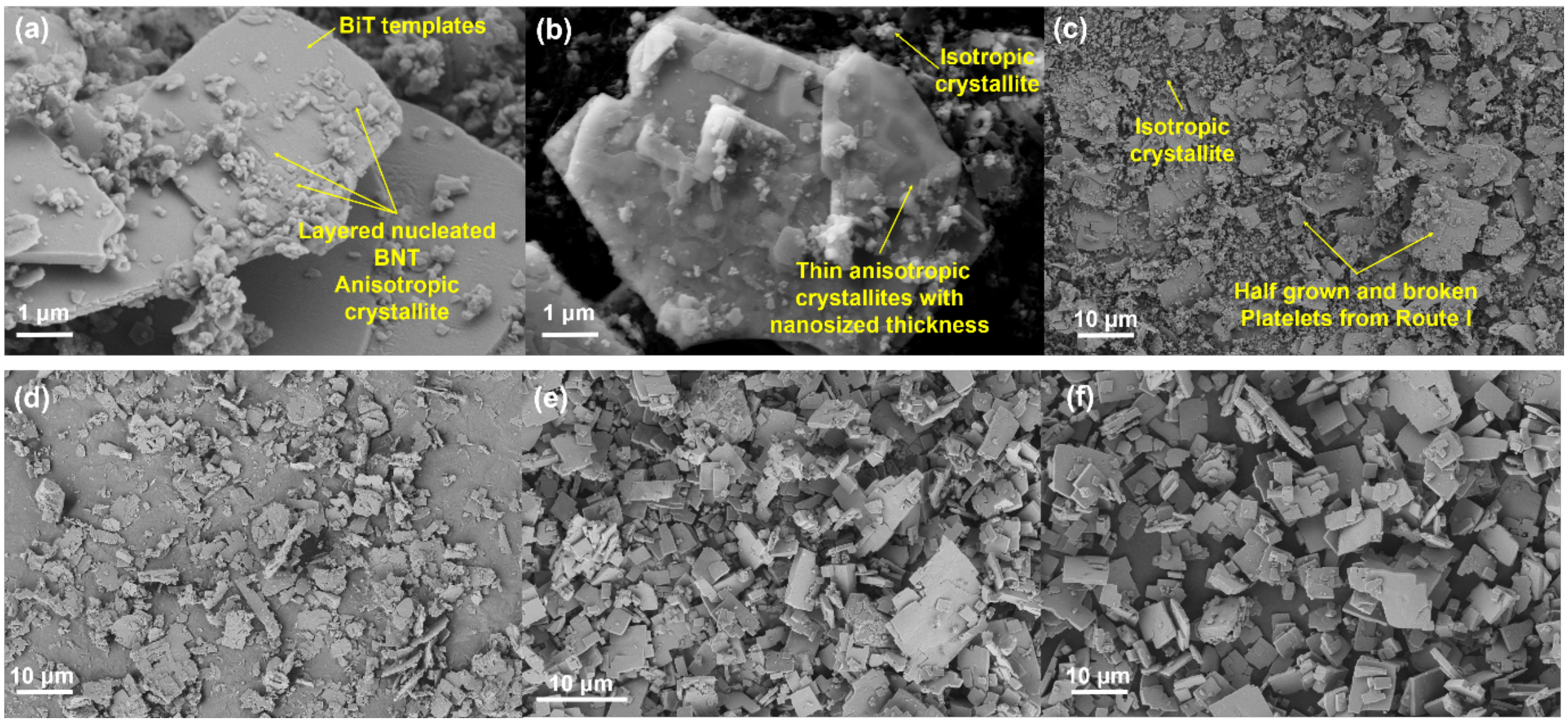

Figure S4. SEM for the BNT systems platelets morphologies that were stopped and cooled at varied temperatures under varied TiO_2 amounts. BNT 5 mol TiO_2 system stopped at (a) 650 °C, (b) 700 °C and (c) 800 °C. BNT 1 mol TiO_2 system stopped at (d) 700 °C, (e) 750 °C and (f) 800 °C. All the mixtures were washed with deionized water and nitric acid.

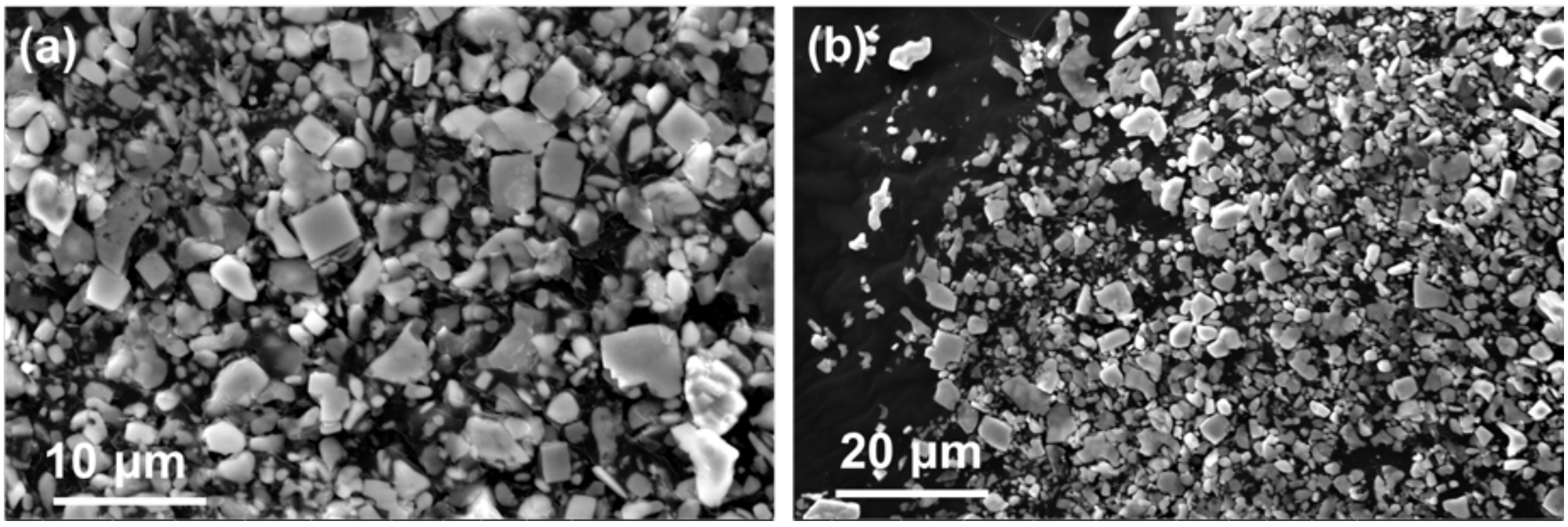

Figure S5. SEM images for BNT powder morphology after acid washing from (a) 5/9 mol Ti amount and 90 mol% excess Na_2CO_3 and (b) 0 mol Ti amount under 90 mol% excess Na_2CO_3.

Table S1. Fitting parameters and quality of the bimodal fitting model for BNT platelets with different TiO_2 contents. The LogNormal fit was defined using $y = y_0 + \frac{A}{\sqrt{2\pi}\cdot w\cdot x} e^{-\frac{\ln\left(\frac{x}{x_c}\right)^2}{2w^2}}$, Peak 1 corresponds to low aspect ratio particles and Peak 2 to high aspect ratio particles.

TiO_2 mol content	5		2.5		1.25		1		0.75		0.55		0	
Peaks (counts)	Peak 1	Peak 2	Peak 1	Peak 2	Peak 1	Peak 2	Peak 1	Peak 2	Peak 1	Peak 2	Peak 1	Peak 2	Peak 1	Peak 2
y_0	Set as 0 during fitting													
x_c	1.26± 0.06	7.9 ± 0.4	1.33 ± 0.05	8 ± 1	1.3 ± 0.1	8.0± 0.4	2.2 ± 0.6	9.8 ± 0.3	1.3 ± 0.1	6.3 ± 0.6	1.3 ± 0.03	6.8 ± 0.6	1.67 ± 0.05	5.3± 0.9
w	0.36 ± 0.06	0.58 ± 0.05	0.20± 0.03	0.7 ± 0.1	0.17 ± 0.07	0.43 ± 0.05	0.5 ± 0.2	0.46 ± 0.04	0.19 ± 0.06	0.5 ± 0.1	0.19 ± 0.02	0.58 ± 0.08	0.36 ± 0.02	0.5 ± 0.1
A	84 ± 5	146 ± 10	26± 2	109 ± 17	15 ± 3	50 ± 5	23 ± 5	191 ± 10	12 ± 2	63± 11	40 ± 2	70 ± 8	60 ± 3	28 ± 7
R^2	0.98		0.91		0.90		0.88		0.80		0.97		0.95	

Table S2. Sizes distribution for all powders.

Samples	10th percentile diameter D_{10} (µm)	Median diameter D_{50} (µm)	90th percentile Diameter D_{90} (µm)	10th percentile Thickness T_{10} (µm)	Median thickness T_{50} (µm)	90th percentile Thickness T_{90} (µm)	10th percentile aspect ratio A_{10} (-)	Median aspect ratio A_{50} (-)	90th percentile aspect ratio A_{90} (-)
BiT	2.48	4.17	7.53	0.23	0.33	0.54	7.54	11.85	14.46
BNT 5 mol TiO_2	0.83	3.31	10.34	0.43	0.83	1.35	1.06	4.85	14.37
BNT 5 mol TiO_2 800°C stopped *(only for anisotropic particles)*	1.53	2.80	4.50	0.12	0.18	0.28	8.0	14.45	28.89
BNT 2.5 mol TiO_2	0.74	2.28	5.85	0.25	0.40	0.76	1.03	5.60	12.66
BNT 1.25 mol TiO_2	0.87	2.80	5.64	0.26	0.39	0.64	1.09	6.80	13.38
BNT 1 mol TiO_2	1.42	3.14	7.78	0.25	0.38	0.71	3.38	8.41	14.51
BNT 1 mol TiO_2 800°C stopped	0.71	2.52	5.53	0.13	0.26	0.48	3.54	10.03	19.12
BNT 1 mol TiO_2 750°C stopped	0.64	2.14	4.62	0.11	0.28	0.48	3.08	7.86	16.29
BNT 1 mol TiO_2 715°C stopped	0.14	0.94	2.81	0.07	0.14	0.29	1.28	6.02	15.50
BNT 0.75 mol TiO_2	1.09	2.45	4.35	0.31	0.53	1.03	1.11	5.33	9.02
BNT 0.55 mol TiO_2	1.03	1.97	4.23	0.33	0.53	0.68	1.07	3.89	8.99
BNT 0 mol TiO_2	1.68	2.20	5.80	0.69	1.41	2.72	1.06	2.09	5.31
BNT-BT 5.51 mol TiO_2	1.74	4.75	9.50	0.73	1.23	1.86	1.21	3.81	9.27
BNT-BT 2.76 mol TiO_2	0.42	2.63	6.55	0.08	0.17	0.42	1.56	16.71	***41.35***

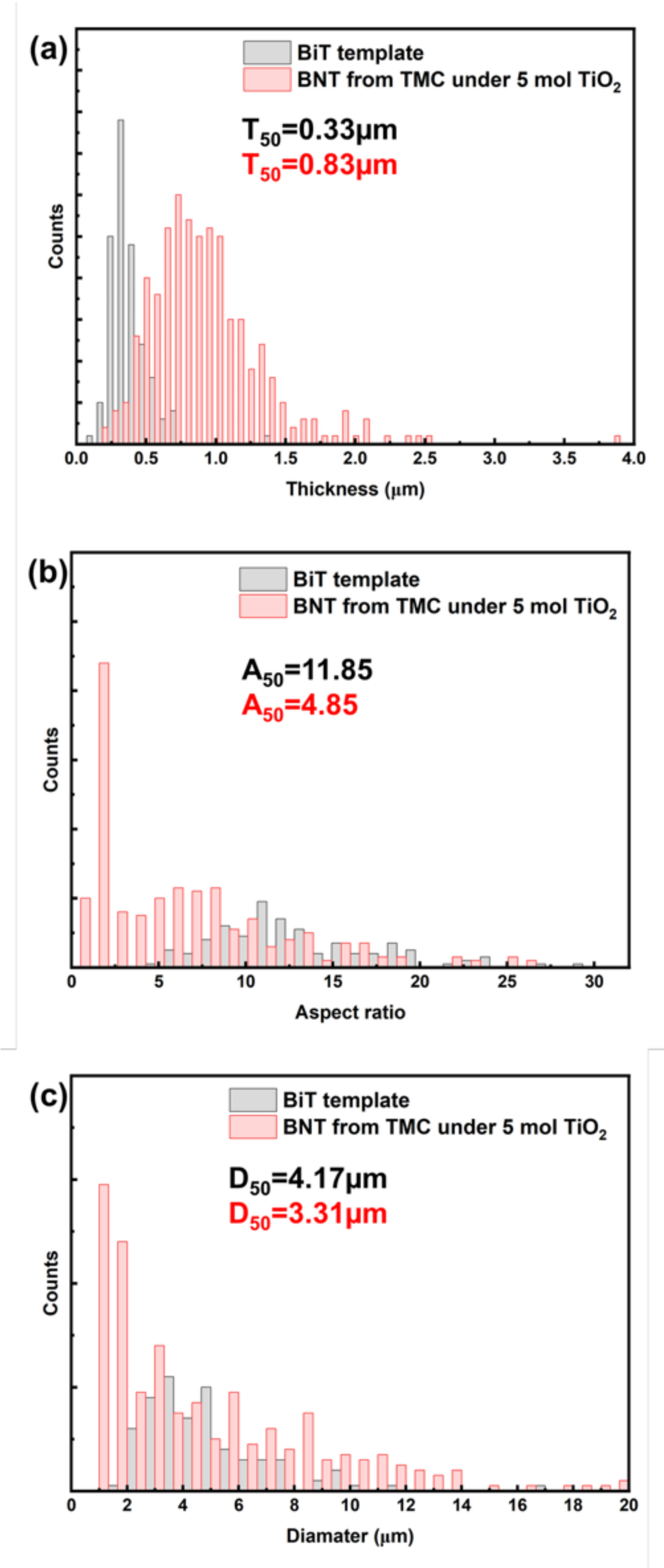

Figure S6. Morphology comparison between BiT templates and BNT platelets synthesised under TMC with 5 mol TiO_2: (a) median thickness T_{50}, (b) median aspect ratio A_{50} and (c) diameter D_{50}, with median reported on each chart.

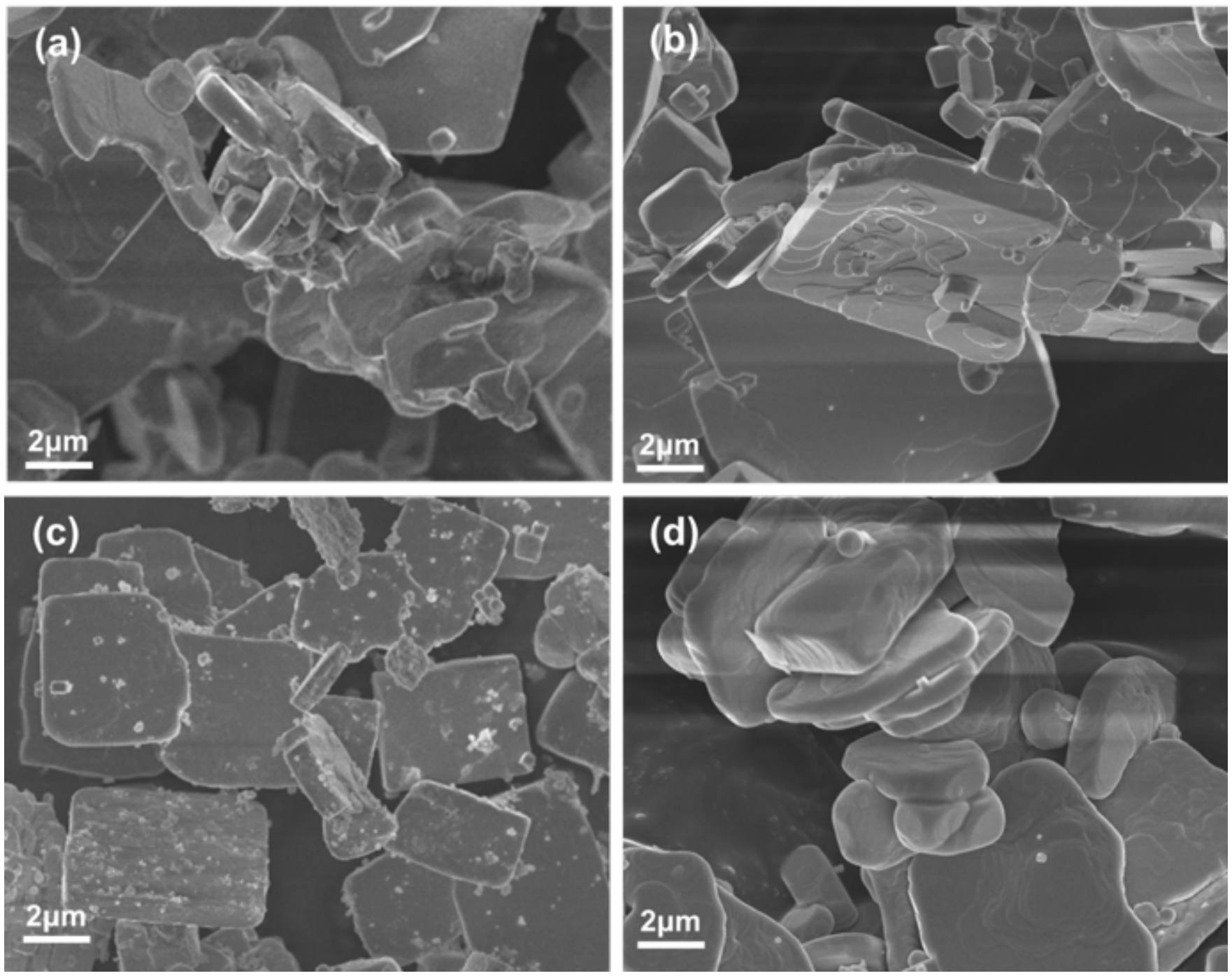

Figure S7. BNT powder fabricated from present 5 mol TiO_2 system with (a) irregular shape and broken size distribution, (b) rough surface facets, (c) tiny equiaxed particles and (d) low aspect ratio platelets.

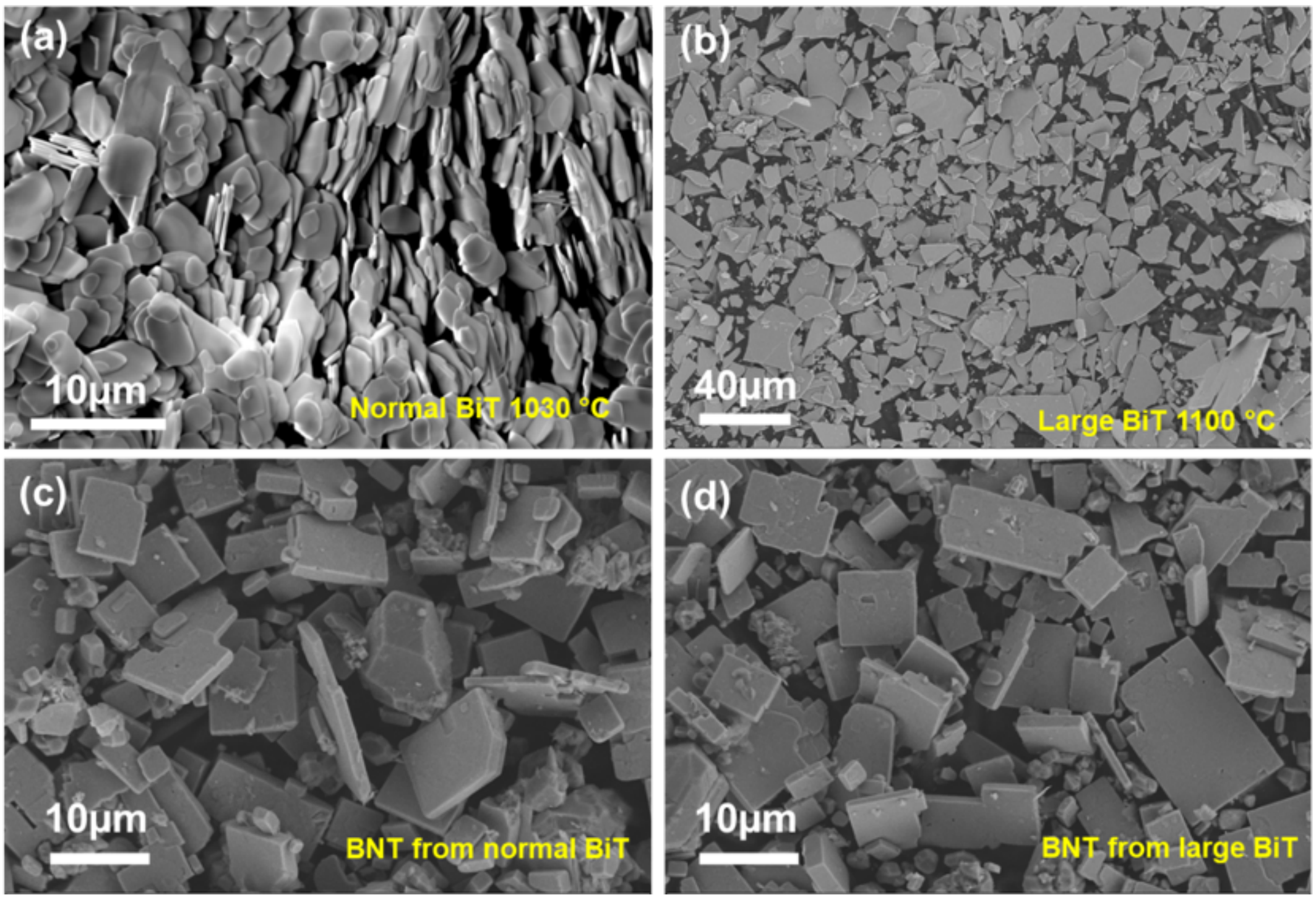

Figure S8. BNT morphology synthesized from different size BiT precursors. (a) Normal BiT synthesised at 1030 °C for 2 hours in molten salt. (b) Larger BiT synthesised at 1100 °C for 2 hours, with diameter ~10-40 μm. (c) The BNT synthesised using normal BiT under 5 mol TiO_2 conditions at 800 °C for 3 hours. (d) BNT synthesised from large BiT under 5 mol TiO_2 conditions at 800 °C for 3 hours.

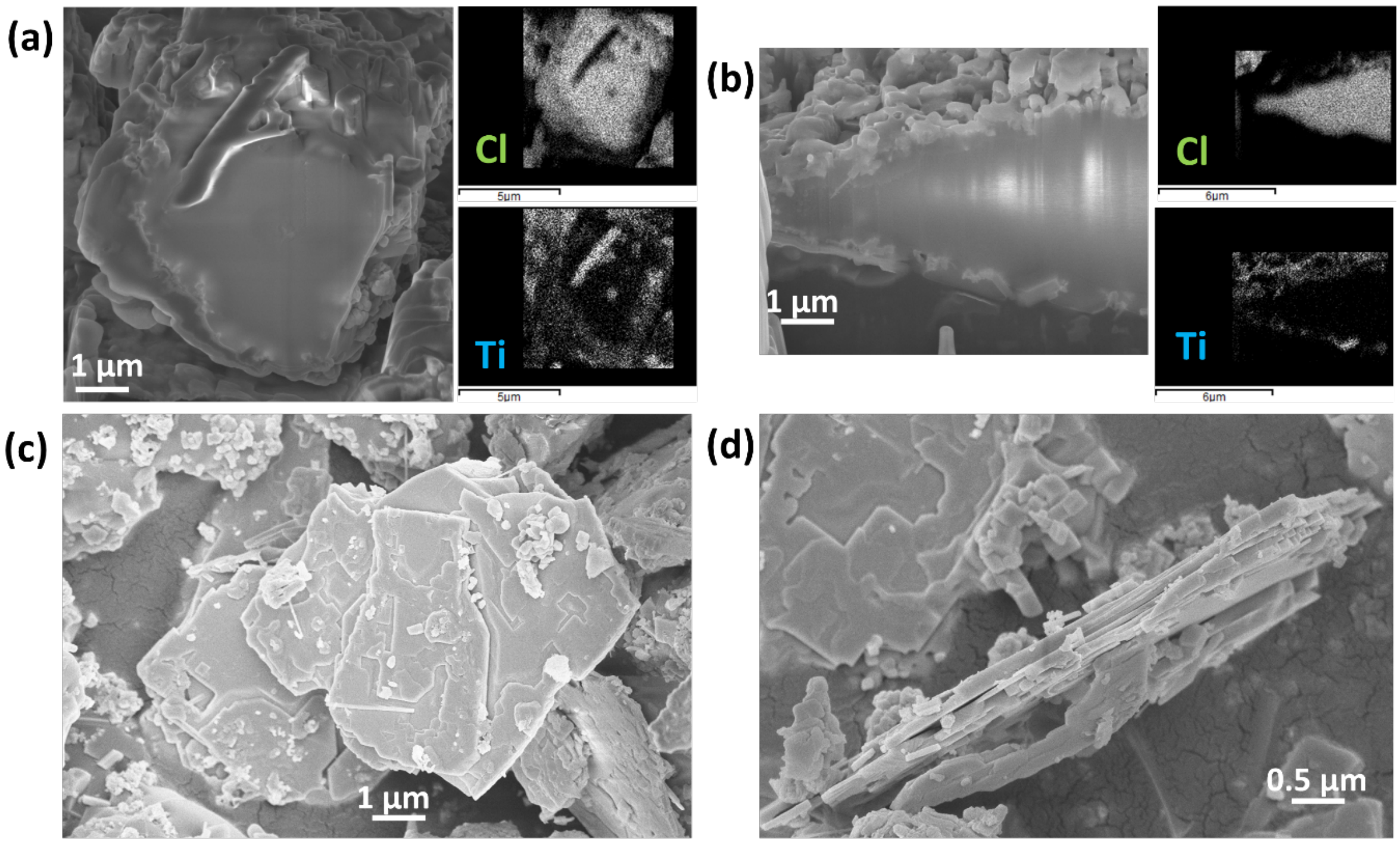

Figure S9. Additional SEM images of the 5 mol TiO_2 powder samples were examined directly after arresting the reaction and cooling at 700 °C. (a) and (b) FIB-milled cross-sections of molten-salt particles, together with the corresponding EDX elemental distributions. (c) and (d) A small fraction of BNT powders from the same batch that were not encapsulated by molten salt, exhibiting fragmented anisotropic crystallites undergoing assembly.

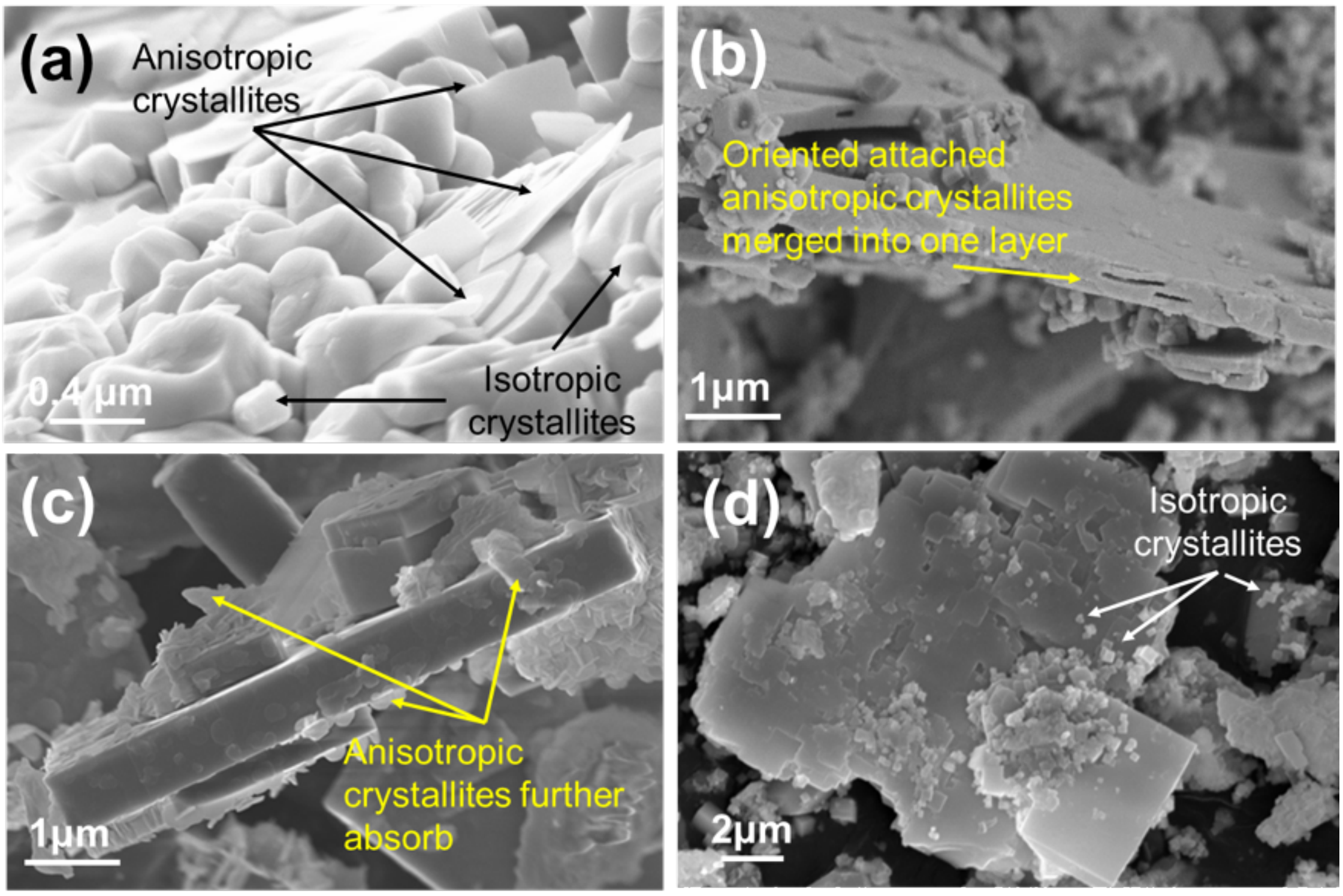

Figure S10. The different mode of anisotropic crystallites and isotropic crystallites observed at BNT grains during grain growth with more zoomed details. (a) After stopping the run at 800 °C oriented attachment between anisotropic crystallites that follow the template shape while the isotropic crystallite assembles on the surface. (b) The oriented attached anisotropic crystallite merged layer-by-layer to get the thickness increasing with layered pores remained. (c) nanoscale anisotropic crystallite oriented attaching to the half-grown particles flat surface. (d) The isotropic crystallites assemble on the half-grown platelets, reducing the aspect ratio.

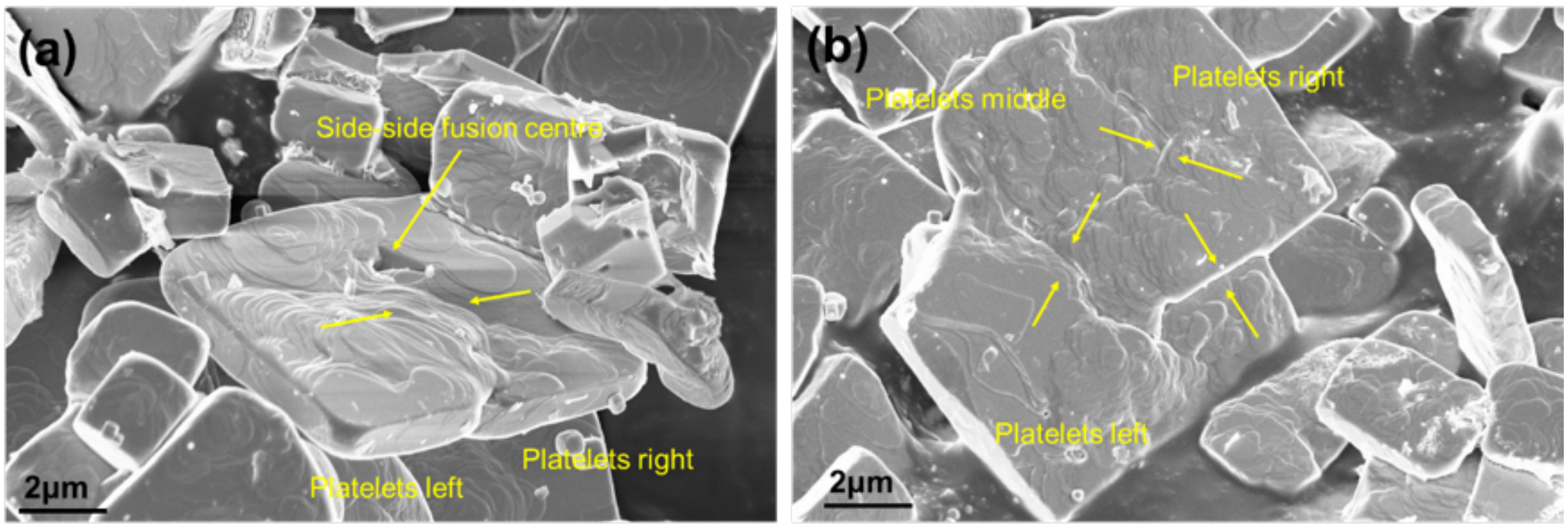

Figure S11. SEM and illustration for BNT platelets at 5 mol TiO_2 edge-edge attaching and recrystallization together. (a) Two half-grown BNT platelets assemble with their edge connection, leaving one central pore that indicate the half-way connection. (b) One more completed edge-edge assemble with 3 platelets, with the connection directions are marked on the images.

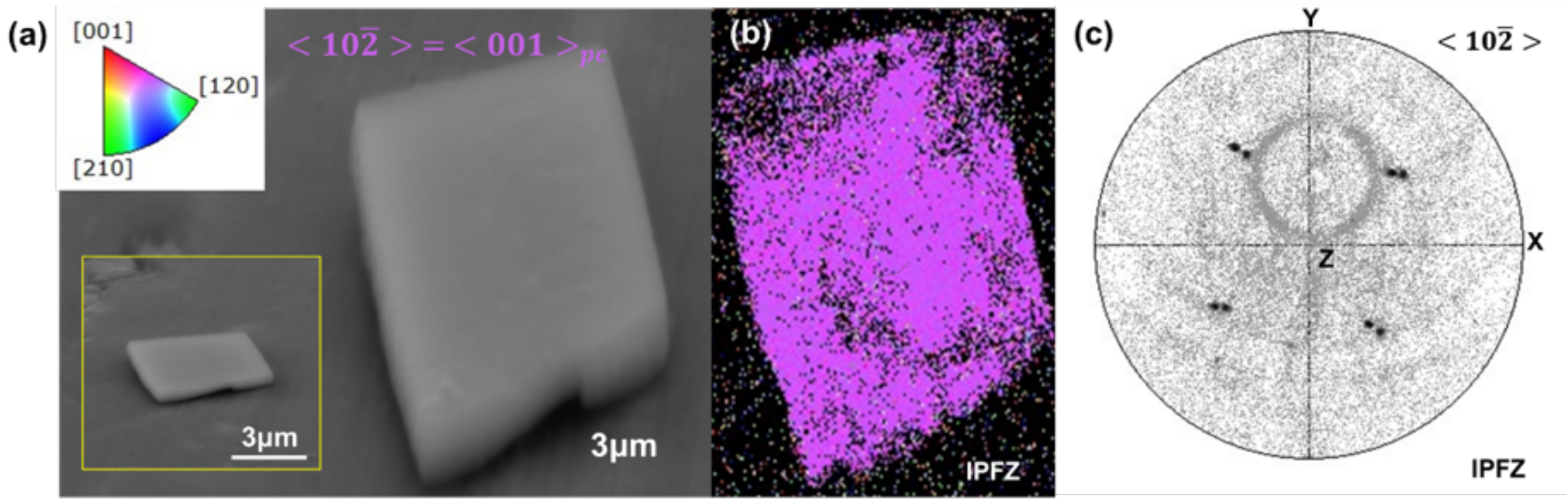

Figure S12. SEM & EBSD pattern for BNT platelets fabricated from 1 mol TiO_2 with (a) SEM for a single crystal under 70° tilting sample substrate and tilting-corrected top view, (b) IPFZ lattice orientation pattern for this crystal and (c) Pole figure at $< 10\bar{2} >$ from the Z-axis.

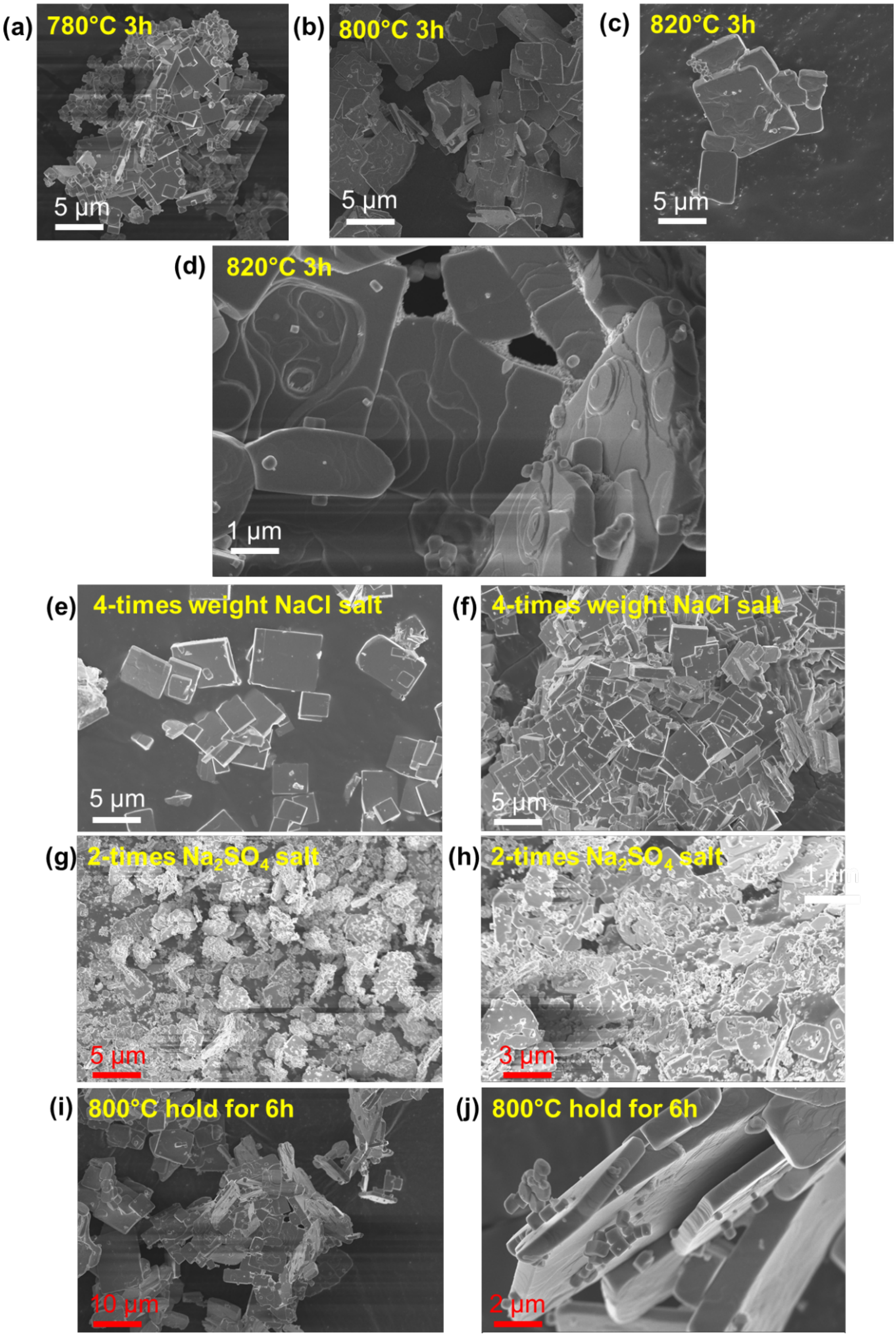

Figure S13. SEM images showing the morphology evolution of BNT powders with BNT 5 mol TiO_2 composition under different reaction conditions: (a) 780 °C, (b) 800 °C, and (c), (d)

820 °C. (e), (f) Morphologies obtained after increasing the NaCl molten-salt content to four times the total mass of the reactants. (g), (h) Morphologies of BNT platelet powders after replacing the molten salt with Na_2SO_4. (i), (j) Morphologies of platelet powders after extending the holding time to 6 h.

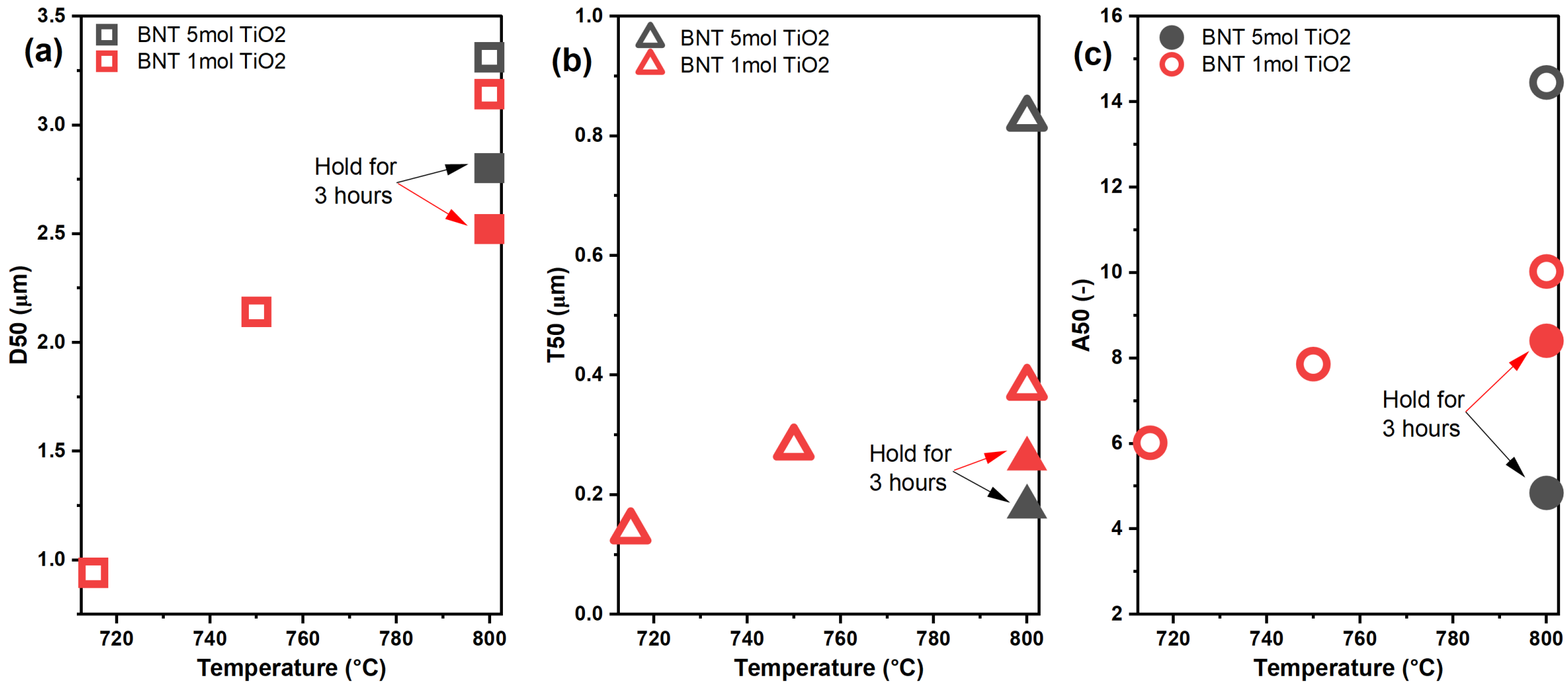

Figure S14. Variations in the median diameter (D_{50}), median thickness (T_{50}), and median aspect ratio (A_{50}) of BNT 5 mol TiO_2 and BNT 1 mol TiO_2 powders under two thermal treatment conditions: immediate cooldown when reaching the target temperature and 3 h holding treatment. Open symbols represent samples for which heating was terminated immediately at the corresponding temperature, whereas solid symbols represent samples subjected to a 3 h holding.

Table S3. Densities for BNT ceramic fabricated from 5 mol and 1 mol TiO_2 powder.

Sample	Relative density (%)
BNT 5 mol TiO_2 ceramic	93% ± 2%
BNT 1 mol TiO_2 ceramic	94% ± 1%

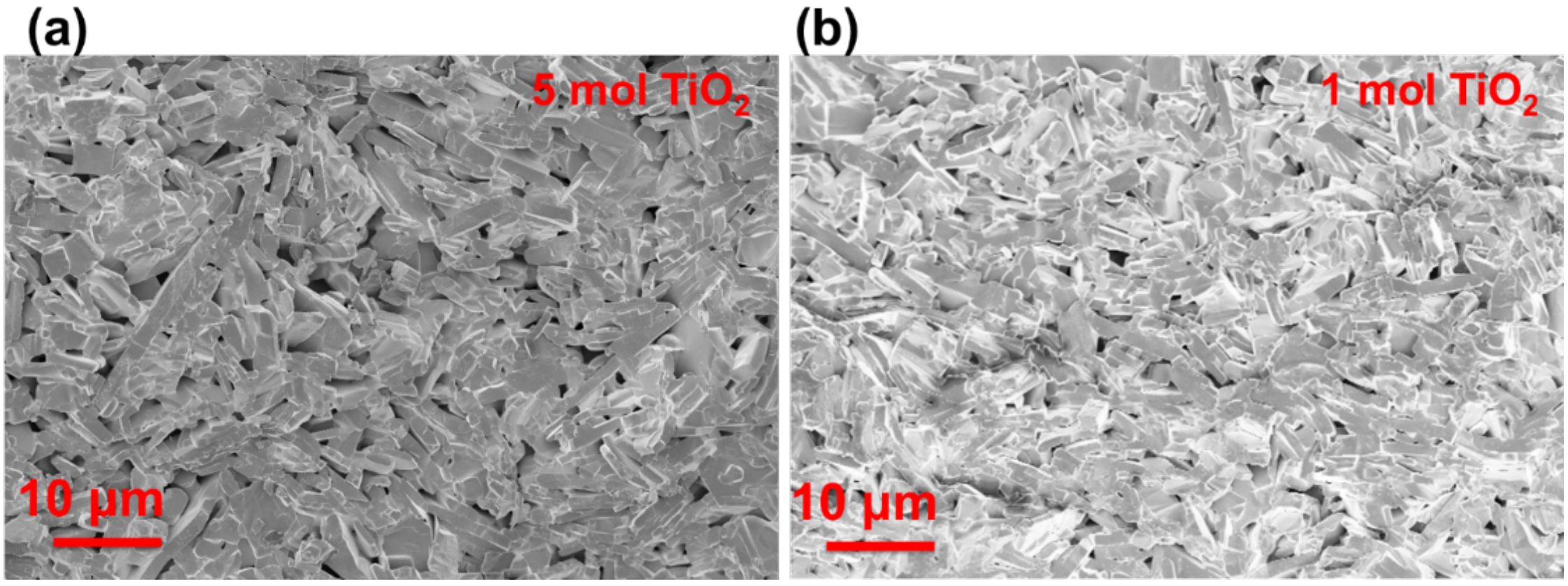

Figure S15. SEM images of the fracture surfaces of ceramics prepared from BNT platelet powders with (a) 5 mol TiO_2 and (b) 1 mol TiO_2.

Table S4. Grain size measurements for ceramics prepared from BNT platelet powders with 5 mol TiO_2 and 1 mol TiO_2, including thickness (T), diameter (D), and aspect ratio (A). The 10th percentile (X_{10}), median (X_{50}), and 90th percentile (X_{90}) values are also included. The data were obtained by statistical analysis of 100-200 grains.

Sample	T_{10} (µm)	T_{50} (µm)	T_{90} (µm)	D_{10} (µm)	D_{50} (µm)	D_{90} (µm)	A_{10} (-)	A_{50} (-)	A_{90} (-)
BNT 5 mol TiO_2 ceramic	0.58	1.01	1.60	1.05	3.91	10.57	1.35	2.81	5.80
BNT 1 mol TiO_2 ceramic	0.48	0.86	1.52	1.84	3.65	8.11	1.88	4.11	7.99

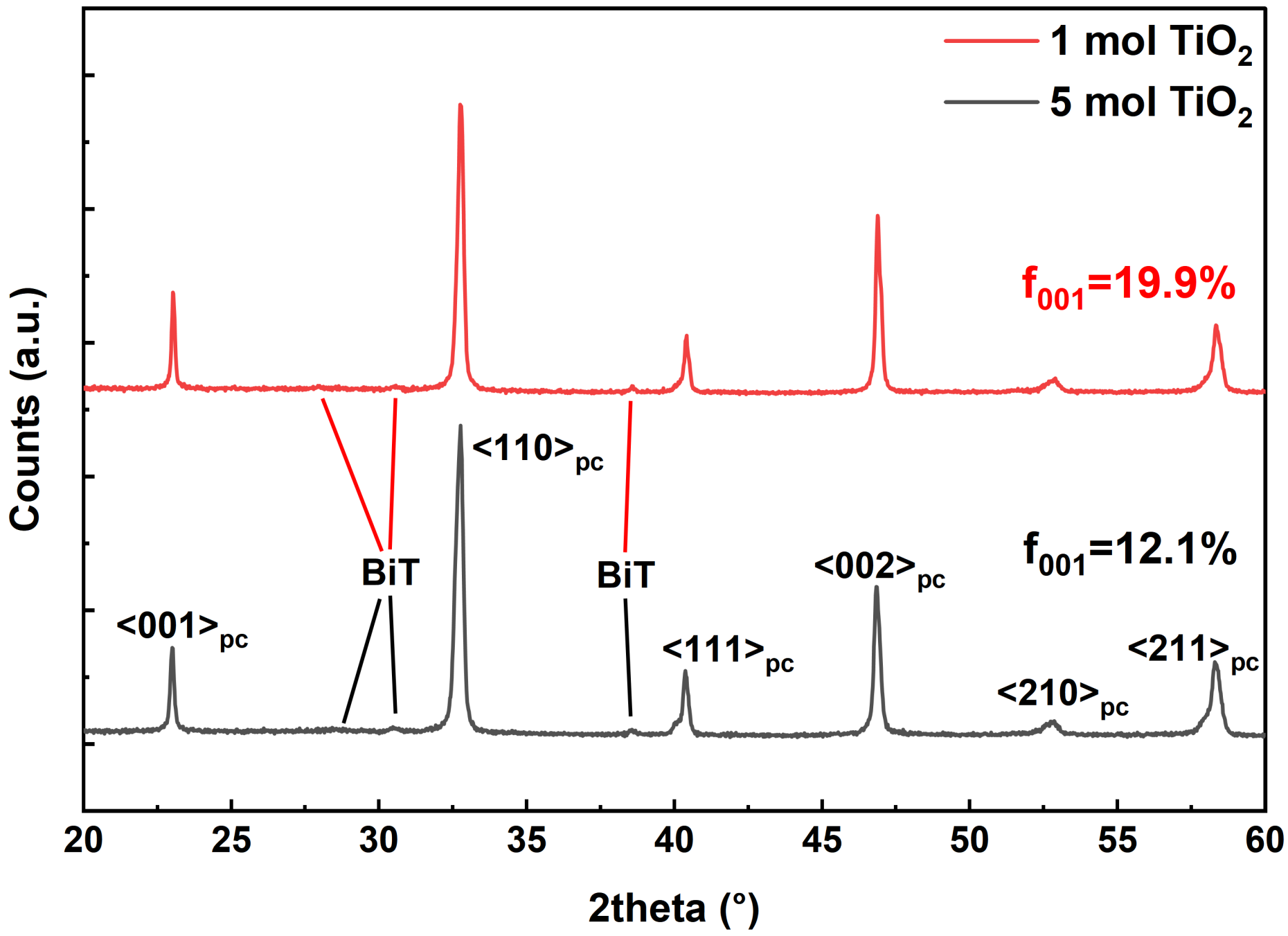

Figure S16. XRD patterns of ceramics prepared from BNT platelet powders containing 5 mol TiO_2 and 1 mol% TiO_2. The BNT reflections are indexed based on the high-temperature cubic phase. The Lotgering factor f_{001} of the [001] was calculated and marked (measured along the conductivity measurement direction). The remaining impurity peaks, mainly attributed to BiT, are also marked.

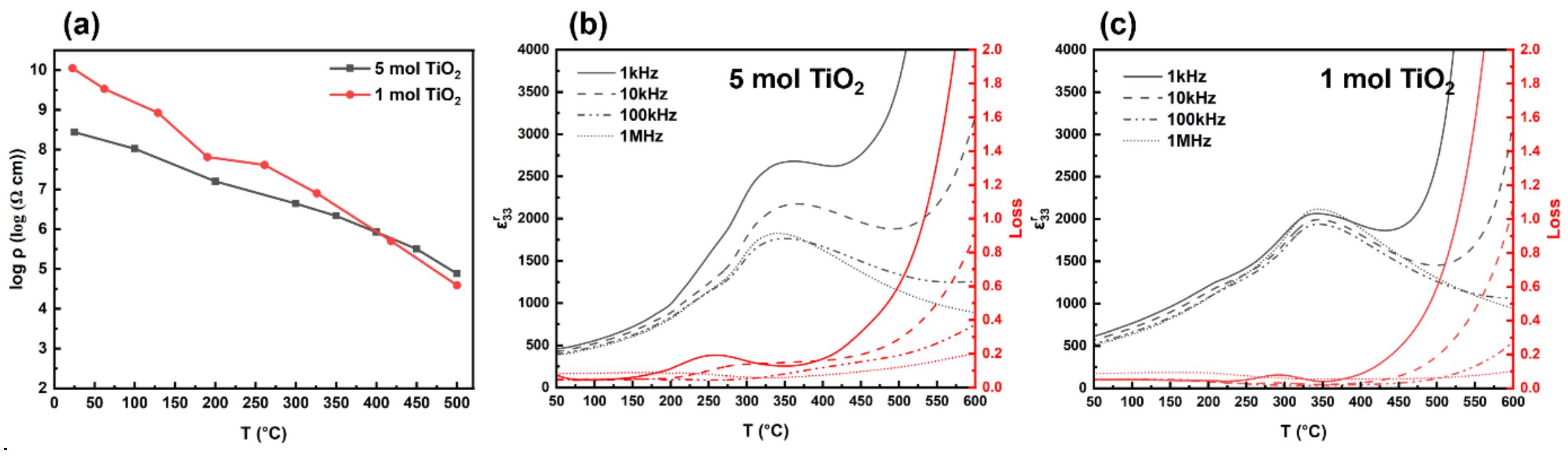

Figure S17. Improved functional properties of BNT ceramics achieved through powder synthesis modification. (a) Temperature-dependent resistivity of BNT ceramics prepared with 5 mol and 1 mol TiO_2 platelets powder. Evolution of the relative permittivity ε_{33}^{r} and dielectric loss as a function of temperature for BNT ceramics prepared by 5 mol TiO_2 (b) and 1 mol TiO_2 (c).

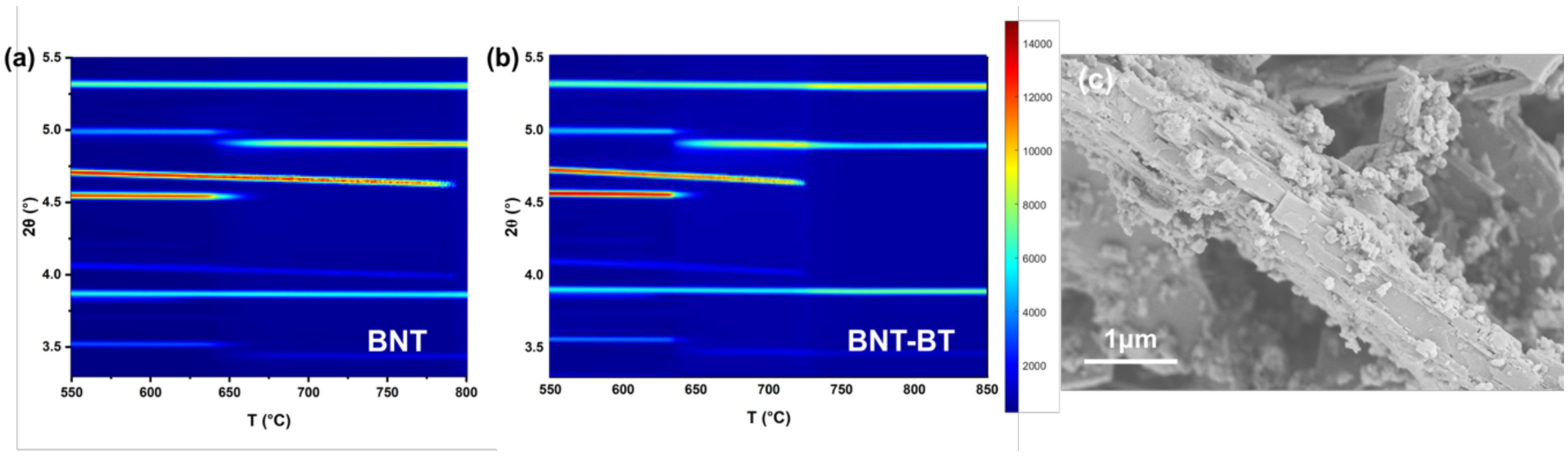

Figure S18. BNT-BT platelets TMC synthesis with a similar reaction and particle growth mechanism as BNT TMC. *In situ* sXRD reconstructed patterns as a function of temperature for (a) BNT with 5 mol TiO_2 and (b) BNT-BT with 5.51 mol TiO_2. (c) SEM morphology for BNT-BT platelets cross-section following thickness direction with 5.51 mol TiO_2 stopped at 680°C where the lattice conversion finished. The BNT-BT platelets also process similar oriented anisotropic crystallites attachment (route I) shown at the thickness direction, and attachment of equiaxed isotropic crystallites on the flat surface (route III), which can also be observed at BNT TMC systems.

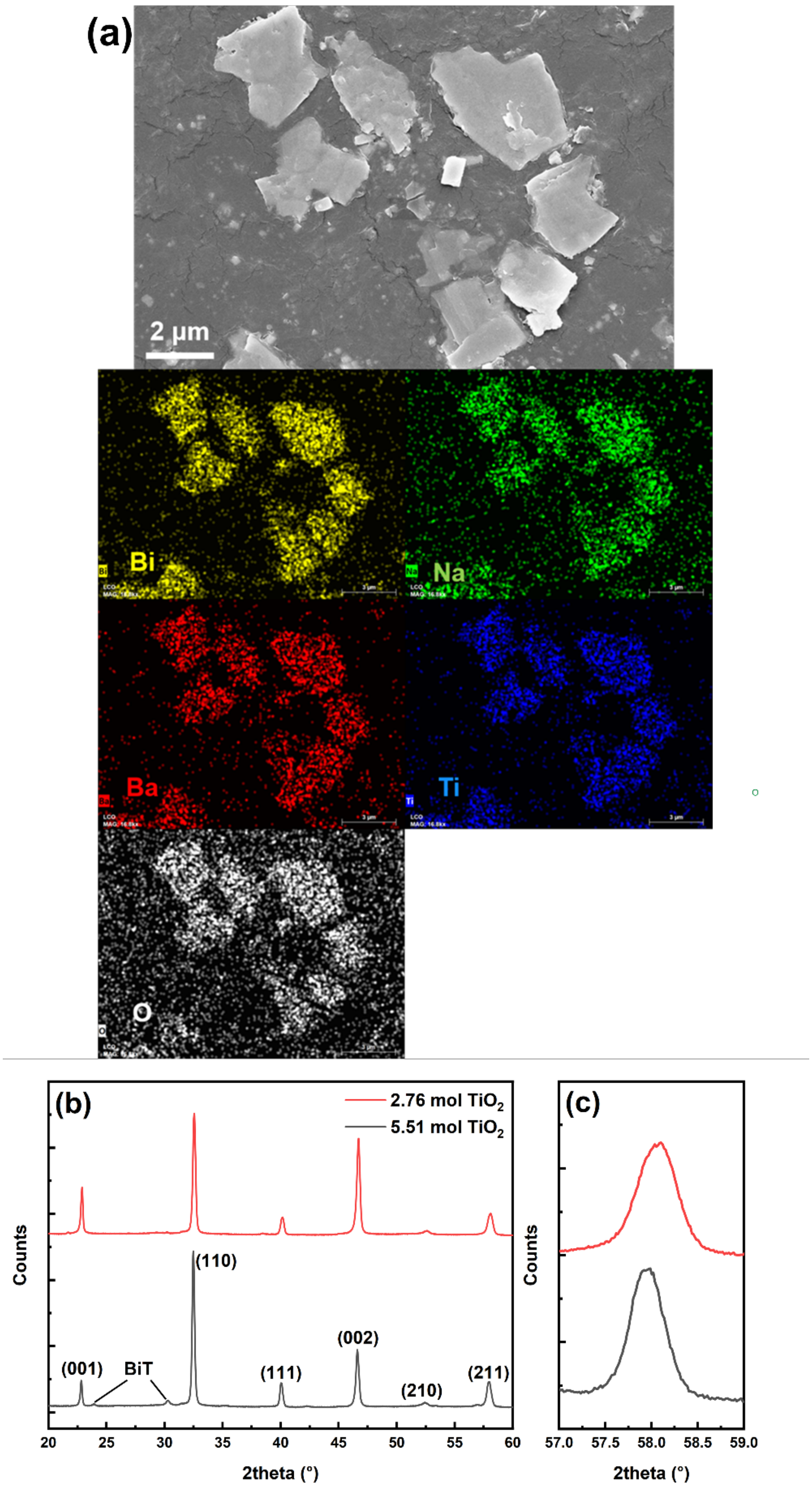

Figure S19. EDX and XRD for BNT-BT platelets synthesized by a novel strategy.

(a) SEM /EDX for BNT-BT synthesized by 50 mol% TiO_2 deficiency 2.76 mol TiO_2

compositions. (b) XRD for the BNT-BT synthesised under 5.51 mol TiO_2 (as stoichiometry) and 2.76 mol TiO_2 (50 mol%). Both exhibit a similar XRD pattern. (c) zoomed in XRD for (211) peak to detail mark the MPB phase peak broadening features, indicating the peaks splitting from the MPD state.

Table S5. Phase ratio and lattice parameters refined from the XRD for the BNT-BT powder fabricated from 5.51 mol TiO_2 and 2.76 mol TiO_2 amounts.

5.51 mol TiO_2						
Phase	Ba Amount	Ratio	a	c	density	wR
R3c	5.5Ba	28.7	5.5013	13.48635	6.005	7.28%
P4bm	7Ba	71.3	5.51098	3.90786	5.745	
2.76 mol TiO_2						
Phase	Ba Amount	Ratio	a	c	density	wR
R3c	5.5Ba	15.3	5.48526	13.45753	6.053	6.528%
P4bm	7Ba	84.7	5.50012	3.90249	6.003	

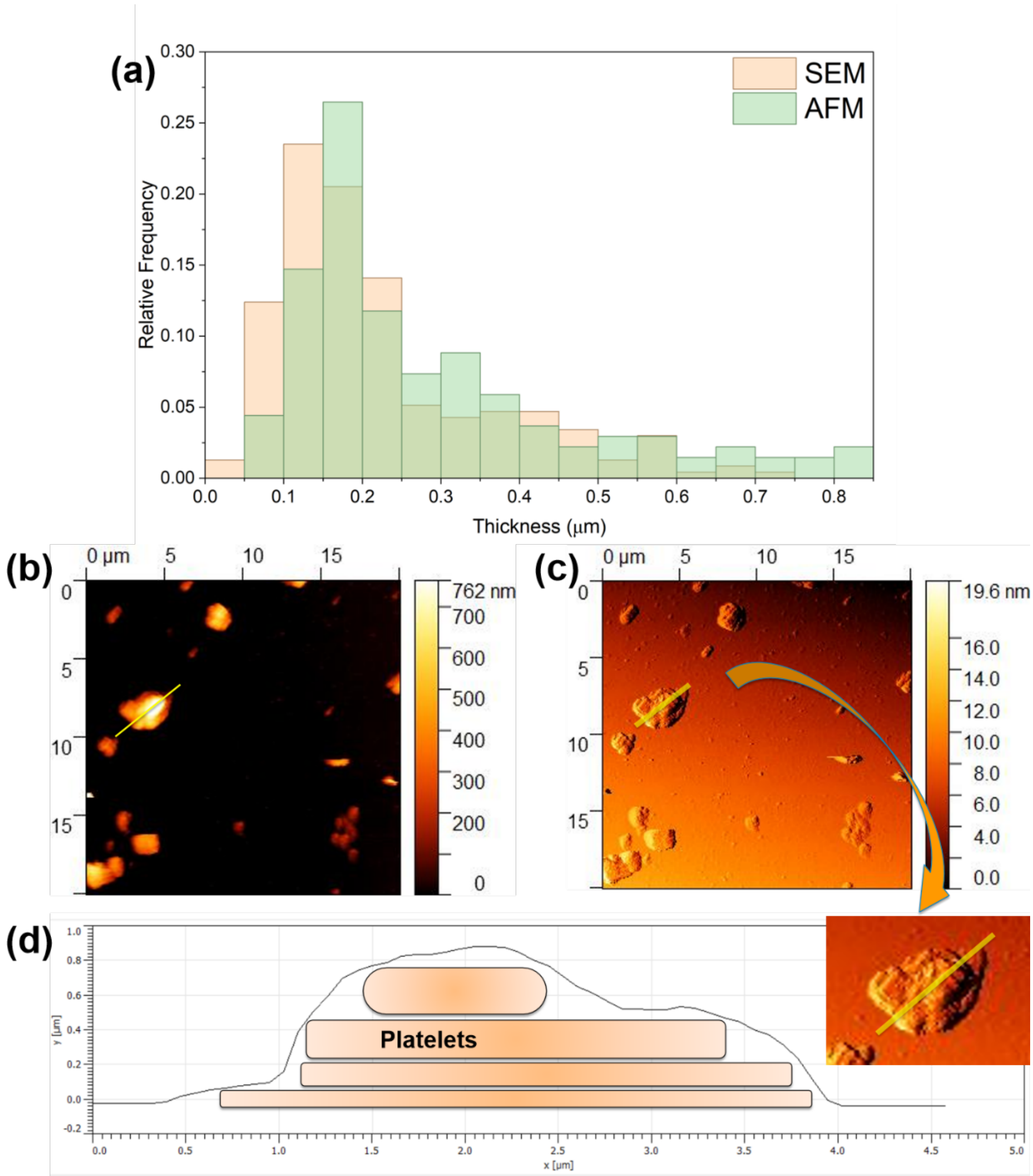

Figure S20. (a) Thickness distribution of the plate-like powders measured by AFM and SEM. For AFM, the powder thickness was measured directly from calibrated AFM height maps. A total of 136 thickness values were collected from all detectable platelets across eight AFM images and plotted in the distribution. (b) Calibrated height mappings of the AFM raw data of BNTBT 2.76 mol TiO_2 powders, (c) the corresponding amplitude trace images provide more direct visualisation of the powder surface morphology in (b). (d) The height profile along the yellow line, showing the variation in

height as a function of the line-scan distance. Surface fluctuations and platelet stacking are illustrated schematically. Inset: Enlarged amplitude trace image of the corresponding region.

Table S6. Statistical summary of the powder thickness.

Samples	10th percentile Thickness T_{10} (μm)	Median thickness T_{50} (μm)	90th percentile Thickness T_{90} (μm)
SEM measured	0.08	0.17	0.42
AFM measured	0.10	0.20	0.58

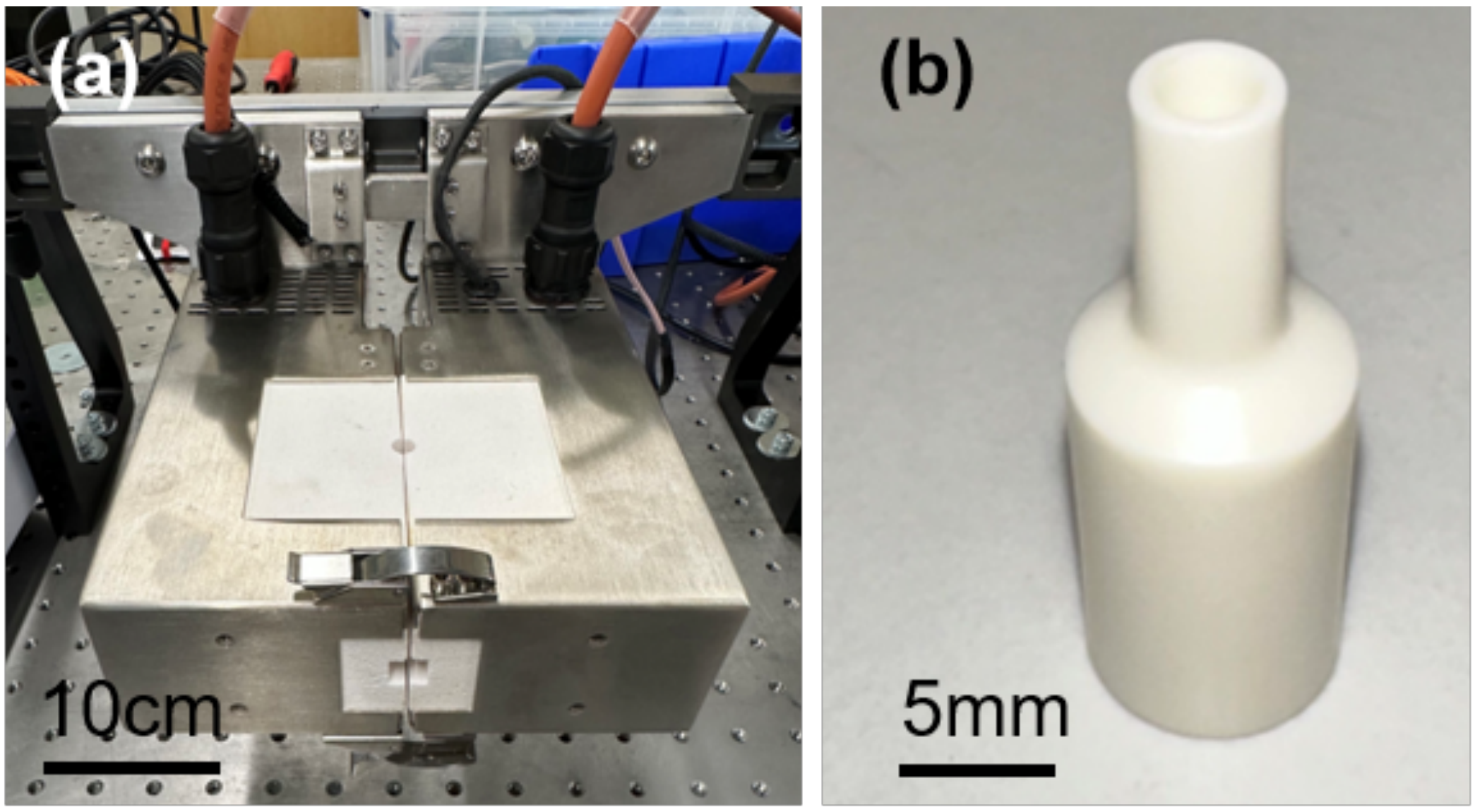

Figure S21. *In situ* synchrotron time-resolved X-ray Diffraction set up: (a) the split furnace SF2149B; (b) the alumina crucible holding the reaction mixtures.

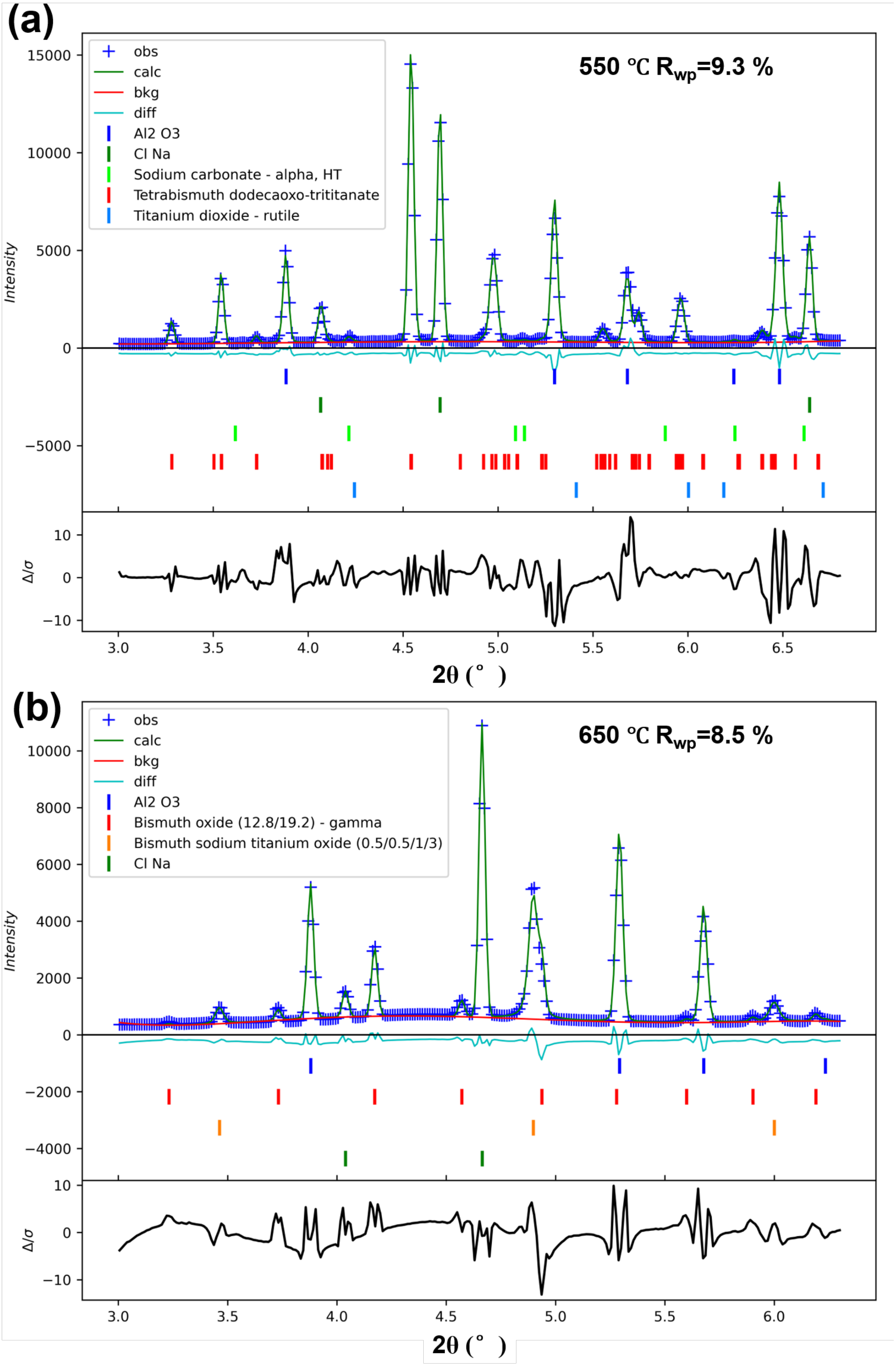

(a)
550 ℃ Rwp=9.3 %
obs
calc
bkg
diff
Al2 O3
Cl Na
Sodium carbonate - alpha, HT
Tetrabismuth dodecaoxo-trititanate
Titanium dioxide - rutile
Intensity
Δ/σ
2θ (°)
(b)
650 ℃ Rwp=8.5 %
obs
calc
bkg
diff
Al2 O3
Bismuth oxide (12.8/19.2) - gamma
Bismuth sodium titanium oxide (0.5/0.5/1/3)
Cl Na
Intensity
Δ/σ
2θ (°)

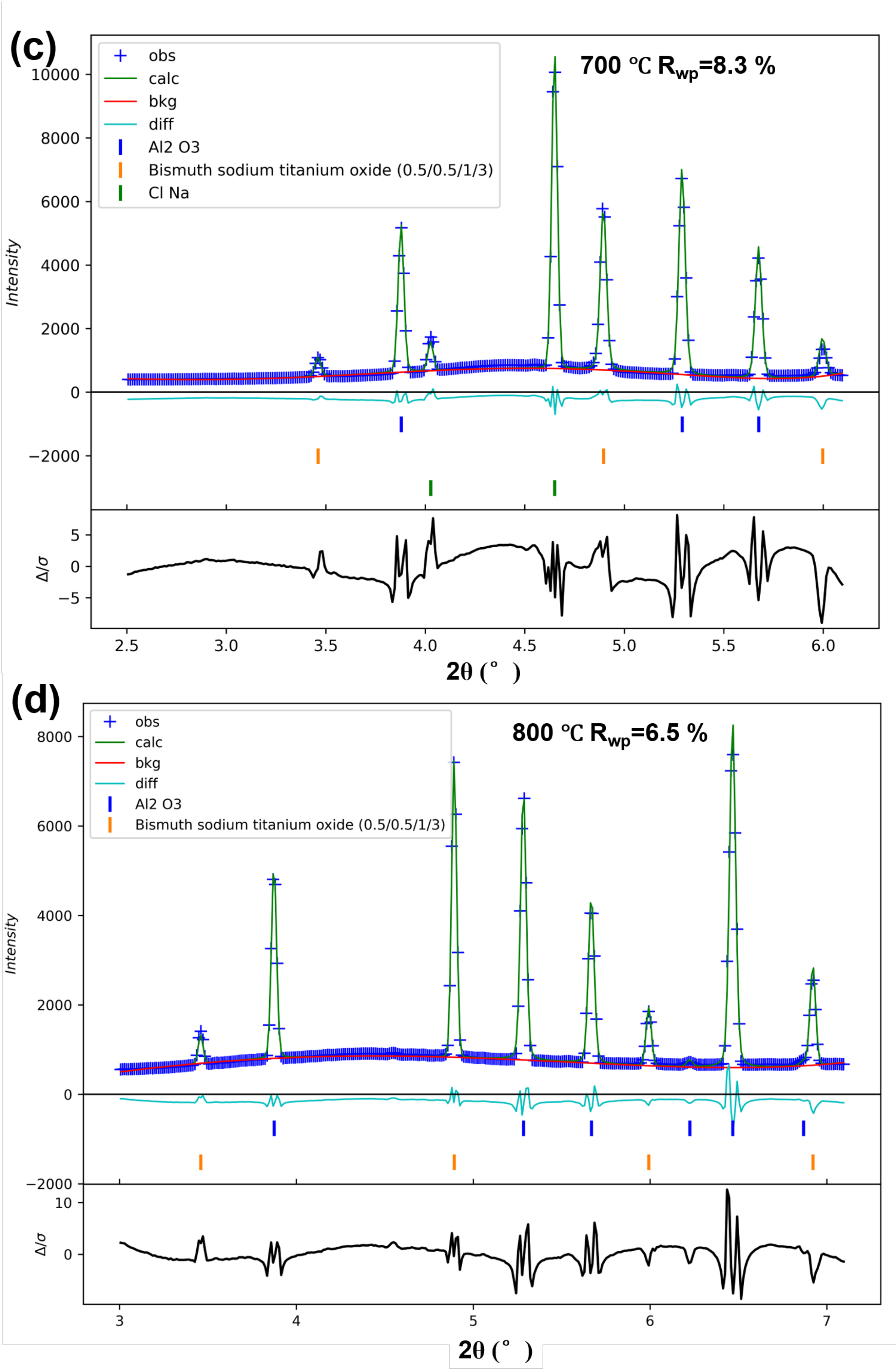
(c)
obs
calc
bkg
diff
Al2 O3
Bismuth sodium titanium oxide (0.5/0.5/1/3)
Cl Na
700 ℃ R_{wp}=8.3 %
Intensity
Δ/σ
2θ (°)
(d)
obs
calc
bkg
diff
Al2 O3
Bismuth sodium titanium oxide (0.5/0.5/1/3)
800 ℃ R_{wp}=6.5 %
Intensity
Δ/σ
2θ (°)

Figure S22. sXRD refinement raw data of 1 mol TiO_2 system after Rietveld refinements including the fitted profile, the difference curve, and the corresponding R_{wp} values. Representative refinements at various temperatures are selected at (a) 550 °C before reaction happened, (b) 650 °C BNT and Bi_2O_3 existed, (c) 700 °C all reagents consumed, and (d) 800 °C NaCl melt and only BNT / Al_2O_3 remained.
